# Ventral midbrain astrocytes display unique physiological features and sensitivity to dopamine D2 receptor signaling

**DOI:** 10.1038/s41386-018-0151-4

**Published:** 2018-07-13

**Authors:** Wendy Xin, Kornel E. Schuebel, Kam-wing Jair, Raffaello Cimbro, Lindsay M. De Biase, David Goldman, Antonello Bonci

**Affiliations:** 1 Intramural Research Program, National Institute on Drug Abuse, National Institutes of Health, Baltimore, MD 21224 USA; 2 Solomon H. Snyder Department of Neuroscience, Johns Hopkins University School of Medicine, Baltimore, MD 21205 USA; 3 Laboratory of Neurogenetics, National Institute on Alcohol Abuse and Alcoholism, National Institutes of Health, Rockville, MD 20852 USA; 4 Department of Rheumatology, Johns Hopkins University School of Medicine, Baltimore, MD 21224 USA; 5 Department of Psychiatry, Johns Hopkins University School of Medicine, Baltimore, MD 21205 USA; 6 Department of Neuroscience, Georgetown University Medical Center, School of Medicine, Washington, DC USA; 7 Department of Psychiatry, University of Maryland School of Medicine, Baltimore, MD USA

**Keywords:** Astrocyte, Cellular neuroscience

## Abstract

Astrocytes are ubiquitous CNS cells that support tissue homeostasis through ion buffering, neurotransmitter recycling, and regulation of CNS vasculature. Yet, despite the essential functional roles they fill, very little is known about the physiology of astrocytes in the ventral midbrain, a region that houses dopamine-releasing neurons and is critical for reward learning and motivated behaviors. Here, using a combination of whole-transcriptome sequencing, histology, slice electrophysiology, and calcium imaging, we performed the first functional and molecular profiling of ventral midbrain astrocytes and observed numerous differences between these cells and their telencephalic counterparts, both in their gene expression profile and in their physiological properties. Ventral midbrain astrocytes have very low membrane resistance and inward-rectifying potassium channel-mediated current, and are extensively coupled to surrounding oligodendrocytes through gap junctions. They exhibit calcium responses to glutamate but are relatively insensitive to norepinephrine. In addition, their calcium activity can be dynamically modulated by dopamine D2 receptor signaling. Taken together, these data indicate that ventral midbrain astrocytes are physiologically distinct from astrocytes in cortex and hippocampus. This work provides new insights into the extent of functional astrocyte heterogeneity within the adult brain and establishes the foundation for examining the impact of regional astrocyte differences on dopamine neuron function and susceptibility to degeneration.

## Introduction

The ventral midbrain, comprised of the ventral tegmental area (VTA) and the substantia nigra pars compacta, houses the majority of dopamine-releasing neurons in the brain. Dopamine (DA) neurons are essential for motivated behaviors, including motor control, reward seeking, and operant learning [1]. Loss of DA neurons or DA signaling, either in rodent models or in the context of Parkinson’s disease, produces many behavioral deficits, including difficulty in initiating movement, cognitive impairments, and depression [2–5]. Although a great deal of effort has gone into defining the physiology and pathology of ventral midbrain DA neurons, very little is known about the physiology of ventral midbrain astrocytes, despite evidence that astrocytes can influence—and are influenced by—the activity of DA neurons [6–8]. Thus, a comprehensive understanding of DA neuron physiology and function will require consideration of ventral midbrain astrocyte physiology and function.

Astrocytes are ubiquitous throughout the central nervous system (CNS). These abundant cells perform numerous critical tissue functions, including buffering extracellular potassium, supporting glutamate homeostasis, regulating synaptic connectivity and plasticity, providing metabolic support to neurons, and participating in injury responses following pathological insults [7, 9–14]. These aspects of astrocyte biology have been extensively studied in hippocampus and cortex and are widely assumed to be equivalent in astrocytes throughout the CNS. However, recent studies provide compelling evidence of regional astrocyte heterogeneity, which likely stems both from adaptation to demands of the local neuronal population and from developmentally programmed differences across distinct pools of neuroendothelial astrocyte progenitors [15, 16]. These findings raise the possibility that differences in astrocyte function contribute to regional differences in neuronal function and vulnerability and argue that astrocyte function must be studied in a region-specific fashion. A recent study examining regional specialization of microglia found pronounced differences between ventral midbrain microglia and microglia in other regions of the basal ganglia [17]. Given the unique features of ventral midbrain neurons that likely exert unique demands on astrocytes, we predicted that astrocytes in the ventral midbrain would be similarly distinct [3, 18, 19]. For example, the tonic firing of DA neurons, combined with their capacity for synchronized burst firing, may require increased potassium buffering capacity, greater metabolic support, and sensitivity to dopamine signaling as a way of gauging DA neuron health and function. Thus, using a combination of histology, RNA sequencing, electrophysiology, and calcium imaging, we carried out a multifaceted characterization of ventral midbrain astrocyte properties. We focused our investigation on the morphology, gene expression, physiology, and calcium activity of astrocytes, as these are fundamental properties of astrocyte biology that define their interactions with surrounding cells. As a point of reference, we also performed parallel experiments in hippocampus and cortex, two non-dopaminergic telencephalic regions where the properties of astrocytes have been thoroughly described.

## Methods

### Mice

All experiments were conducted in accordance with the National Institutes of Health Guide for the Care and Use of Laboratory Animals. Animals were housed on a 12 h light/dark cycle and given ad libitum access to food and water. *Aldh1l1-eGFP* mice were obtained from the Mutant Mouse Resource and Research Centers (MMRRC; Stock #011015-UCD). *GFAP-cre* animals were originally obtained from Jackson Laboratory (Stock No. 012887). *Aldh1L1-eGFP* mice and *GFAP-cre* mice were maintained on FVB and C57Bl6 backgrounds, respectively; heterozygotes were used for all experiments.

### Immunohistochemistry

Male and female *Aldh1L1-eGFP* mice, aged 2–3 months, were anesthetized with Euthasol and perfused transcardially with phosphate-buffered saline (PBS) and 4% paraformaldehyde and post-fixed in 4% paraformaldehyde for 4 h. Then, 60 µm sections were collected on a vibratome (Leica VT1000). Sections were permeabilized and blocked with 0.02% Triton-100 and 5% donkey serum for 1 h at room temperature (RT), then incubated with primary antibodies (see Supplementary Table 1 for list of primary antibodies) overnight at 4 C, and secondary antibodies (donkey Alexa 488, 594, or 647; Jackson ImmunoResearch) the next day for 2 h at RT.

### Confocal image acquisition and analysis

Sections mounted on glass microscope slides with Mowiol mounting medium containing 4′,6-diamidino-2-phenylindole were imaged with an upright confocal laser scanning microscope (Olympus FV-1000). For VTA, images were taken between the interpedoncular nucleus and the substantia nigra pars compacta, where DA neuron density (identified by tyrosine hydroxylase staining) is highest. For hippocampus, images were taken from the ventral CA1. For cortex, images were taken from the entorhinal cortex. All three regions were sampled from the same coronal slice, at ~-5.0 mm from Bregma. The size of regions sampled was 528 µm by 528 µm. Density was determined using the cell counting function on ImageJ. Percent tissue coverage was calculated from a maximum intensity projection generated in ImageJ from a *Z*-stack captured at 1 µm intervals. Mean pixel intensity of the dimmest identifiable cell processes was determined by averaging values across 15 locations for each image and used as the threshold for calculating % pixels occupied by enhanced green fluorescent protein (eGFP) processes. Soma size was measured from the same maximum intensity projections in which manually drawn regions of interest (ROIs) were used to calculate area of cell bodies. There was no significant difference between male and female mice in the observed measures.

For VGluT staining and synapse density analysis, confocal images were taken of sections from P60 CX3CR1-GFP animals immunostained with primary antibodies for VGluT1, VGluT2, and VGluT3. Regions sampled are identical to what was described above. For analysis, three optical sections from the same *Z*-stack image (imaged at a 0.3 µm interval) were collapsed (maximum intensity, Fiji) and analyzed using the Spots tool in Imaris v. 7.7.1. For each animal, puncta densities were calculated using images taken from two separate slices and averaged. Total density of VGluT puncta represents the sum of VGluT1, VGluT2, and VGluT3 puncta calculated for each animal.

### Tissue dissociation and flow cytometry

Due to the high cost associated with flow cytometry and RNA sequencing, we used males exclusively in these experiments to minimize possible variability resulting from sex differences. Male *Aldh1L1-eGFP* mice, aged 2–3 months, were deeply anesthetized with Euthasol and perfused transcardially with sodium-substituted *N*-methyl-d-glucamine artificial cerebral spinal fluid (NMDG ACSF) containing, in mM, 92 NMDG, 20 HEPES, 25 glucose, 30 NaHCO_3_, 1.2 NaH_2_PO_4_, 2.5 KCl, 5 sodium ascorbate, 3 sodium pyruvate, 2 thiourea, 10 MgSO_4_, and 0.5 CaCl_2_ (pH 7.35, ~300 mOsm, saturated with 95% O_2_/5% CO_2_). Brains were extracted and sectioned on a vibratome (Leica VT-1200) in NMDG ACSF. Two 200 μm horizontal sections containing ventral midbrain were collected. Ventral midbrain, cortex, and hippocampus were identified via white matter landmarks, microdissected, minced, and transferred to microtubes containing ice-cold Hank’s Buffered Salt Solution (no Ca^2+^/Mg^2+^). For ventral midbrain, ~2 mm square sections lateral to the mammillotegmental tract were dissected, which contained the VTA and the substantia nigra pars compacta. For cortex, ~3 mm square sections containing layers 1–6 of entorhinal cortex were dissected. For hippocampus, ~3 mm sections containing CA3 and CA1 regions of ventral hippocampus were dissected. For all three regions, sections were collected at approximately the same anterior–posterior position (~−4.5 to −6.5 mm from Bregma; see Figure S2). Tissue was dissociated into a single-cell suspension using the Miltenyi Neural Tissue Dissociation Kit (version ‘P’). Briefly, tissue was enzymatically digested for 30 min at 37 °C and manually triturated using fire-polished glass Pasteur pipets with progressively smaller diameter openings (final pipet ~0.4 mm diameter) [20]. Cell suspensions were pelleted and resuspended in PBS for sorting via flow cytometry.

Samples were sorted using a BD Biosciences FACS Aria I cell sorter into 50 µL of Arcturus PicoPure RNA isolation kit extraction buffer. Cell suspensions from wild-type tissue were used to establish GFP-negative gates. Astrocytes were identified based on fluorescein isothiocyanate (FITC) channel intensity (i.e., eGFP expression; Figure S2). Using tissue microdissected from individual animals, we collected an average of 145 ± 61 cells from the ventral midbrain, 1119 ± 512 cells from the cortex, and 427 ± 301 cells from the hippocampus (per animal). RNA isolation from fluorescence-activated cell sorting (FACS)-purified astrocytes was performed using the Arcturus Picopure RNA isolation kit.

### RNA sequencing and analysis

A pipeline for low-input whole-transcriptome RNA sequencing from microdissected, FAC-sorted cells was developed; details have been recently published [17]. For hippocampus, 6 samples were sequenced with a total number of 69,125,533 mapped reads; for cortex, 6 samples with 62,375,749 mapped reads; and for the ventral tegmental area, 5 samples with a total of 72,053,498 mapped reads. All datasets passed quality control assessment using boxplot, volcano, and AB plot analysis. Group A versus group B statistical comparisons with associated *p* values and fold changes were generated by CLC EDGE test algorithms. Empirical analysis of Digital Gene Expression (EDGE) [21] is similar to Fisher’s exact test and was developed specifically for count data where many features are analyzed simultaneously across few biological replicates. Unless otherwise stated, genes were considered differentially expressed if EDGE test *p* < 0.05. RNA sequencing data have been submitted to the National Center for Biotechnology Information (NCBI) Sequence Read Archive (SRA) as project number SRP153363.

Genes were considered expressed in a group if mean RPKM (reads per kilobase million) was >2 and normalized SEM (SEM/mean RPKM) was <0.5 (see Supplemental Table 2 for lists of expressed genes). Functional annotation of gene lists was carried out using Ingenuity Pathway Analysis (IPA, QIAGEN) supplemented by manual survey of current astrocyte scientific literature. Functional annotation of the most conserved and the most differentially expressed genes was carried out as follows: those genes with p < 0.01 in at least one pairwise comparison were considered the most differentially regulated (total of 520 genes; RPKM greater than 2, SEM < 0.5 in the most highly expressing region). To select an equal number of highly conserved genes, those genes that were expressed (RPKM greater than 2, SEM < 0.5) in all regions and had no significant *p* value in any pairwise comparison were ranked by average *p* value across all pairwise comparisons. The 520 genes with the largest average *p* value were considered the most conserved genes (average *p* value was >0.45). These lists of most conserved and most differentially regulated genes were imported into IPA and functional families were ranked according to the proportion of genes that were found to be highly conserved or highly differentially regulated. For generation of heat maps, all genes that were associated with astrocyte calcium dynamics or astrocyte electrophysiological membrane properties, either via functional annotation using IPA or via manual survey of recent astrocyte literature, and that were expressed (RPKM greater than 2, SEM < 0.5) by astrocytes in at least one region, were considered. Fold change values generated by CLC EDGE algorithm for these genes were uploaded into CIMminer (NCI/NIH) and heat maps were generated using quantile binning. Venn diagrams were generated using Venny^2.1^ (https://bioinfogp.cnb.csic.es/tools/venny/).

### Electrophysiology and biocytin cell filling

*Aldh1L1-eGFP* mice aged 2–3 months, male and female, were anesthetized with Euthasol and perfused with ice-cold NMDG ACSF (as described above). Horizontal slices 150 μm in thickness were collected using a Leica VT-1200 vibratome, then transferred to a recovery chamber containing NMDG ACSF maintained at 32 °C. After 10 min, slices were transferred to a holding chamber maintained at RT; recordings were made at least 1 h after slicing. ACSF used for holding slices was identical to NMDG ACSF, except it contained 92 mM NaCl instead of NMDG and 1 mM MgCl_2_ and 2 mM CaCl_2_. ACSF used to perfuse the slice during recording contained (in mM): 125 NaCl, 2.5 KCl, 1.25 NaH_2_PO_4_, 1 MgCl_2_, 2.4 CaCl_2_, 26 NaHCO_3_, and 11 glucose. All ACSF solutions were saturated with 95% O_2_/5% CO_2_. During recording, slices were kept in a recording chamber at RT and visualized with an upright microscope (Olympus BX51). Slices were perfused with ACSF (2.5 mL/min) using a peristaltic pump (WPI). Astrocytes were identified by eGFP expression and large membrane conductance. VTA astrocytes were recorded in the area immediately medial to middle temporal (MT); hippocampal astrocytes were recorded in the stratum radiatum of the CA1; cortical astrocytes were recorded in superficial layers of entorhinal cortex. Whole-cell recordings were performed with borosilicate glass patch pipettes (3–5 MΩ resistance) filled with an internal solution containing (in mM): 115 potassium methylsulfate, 20 NaCl, 1.5 MgCl_2_, 10 BAPTA, 10 sodium phosphocreatine, 4 Mg-ATP, and 0.4 Na_2_-GTP (pH 7.35, 295 mOsm). Whole-cell voltage clamp recordings were made using a MultiClamp 700B amplifier (10 kHz digitization and 1–2 kHz low-pass Bessel filter) with pClamp 10.3 software (Molecular Devices). Throughout the recordings, series resistance was monitored (0.1 Hz) with a 5 mV hyperpolarizing step. Cells in which series resistance changed more than 20% during the recording were excluded from analysis. To measure gap junction coupling, biocytin (1.5 mg/mL) was added to the internal solution prior to recording. Cells were held for 25 min to allow for diffusion of biocytin through gap junctions. After recording, slices were post-fixed and stained with antibodies against streptavidin and imaged as described above. There was no significant difference in the observed measures between male and female mice.

### Calcium imaging

*GFAP-cre* mice aged 2–3 months, male and female, received unilateral 0.5 µL stereotaxic injections of cre-dependent GCaMP6m (AAV1.CAG.Flex.GCaMP6m.WPRE.SV40; titer 6.46^e12^ GC/mL; Penn Vector Core) into either the VTA (antero-posterior (AP) −3.10, medio-lateral (ML) −1.38, dorso-ventral (DV) −4.87) or the ventral hippocampus (AP −3.60, ML 2.85, DV −4.70). At 4 weeks after injection, mice were anesthetized with Euthasol and transcardially perfused with ice-cold NMDG ACSF (described above). The 200 μm thick horizontal slices were collected using a Leica VT-1200 vibratome. Slices were recovered as described above and imaged using a custom two-photon laser scanning microscope, controlled by ScanImage written in MATLAB. Ventral midbrain astrocytes were visualized in the area adjacent to MT; hippocampal astrocytes were visualized in the CA1; cortical astrocytes were visualized in the entorhinal cortex. GCaMP6 signal was excited at 910 nm with a Ti:Sapphire laser (Spectra-Physics Mai Tai) and detected via PMT through a dichroic mirror (Sutter Instrument; ET525/50m filter, Chroma). Time-lapse images were acquired at 1 Hz, 512 × 512 pixels per frame, using a 63× water immersion objective (Zeiss). Microscope was fitted with a custom perfusion chamber. Slices were perfused in regular ACSF saturated with 95% O_2_/5% CO_2_. Where indicated, drugs were bath applied at the following concentrations: norepinephrine 40 μM, glutamate 1 mM, quinpirole 10 μM, sulpiride 300 nM, lidocaine 500 μM, kyneurinic acid 100 μM, picrotoxin 100 μM, and CGP35348 1 μM. There was no significant difference in the observed measures between male and female mice.

Image analysis was performed in ImageJ. XY drift was corrected using the ImageJ plugin StackReg; slices with *Z* drift were excluded. Analysis was restricted to the somatic regions of morphologically identified astrocytes, to exclude the possibility of any contributions from neuronal calcium signals. Astrocyte somas were manually defined as ROIs using the ROI tool. Intensity measurements for each cell were extracted and background subtracted. Values were normalized to baseline as *Z*-scores. Raster plots were generated in MATLAB.

### Statistical analysis

Data are presented as mean ± SEM. Graphs were generated using Graphpad Prism software and Microsoft Excel. Statistical significance was determined with one-way analysis of variance (ANOVA) and post hoc Bonferroni tests unless otherwise indicated. For sequencing data, differentially expressed genes were determined using the EDGE test within the CLC Genomics Workbench software.

## Results

### VTA astrocytes are morphologically distinct from telencephalic astrocytes

**Fig. 1:**
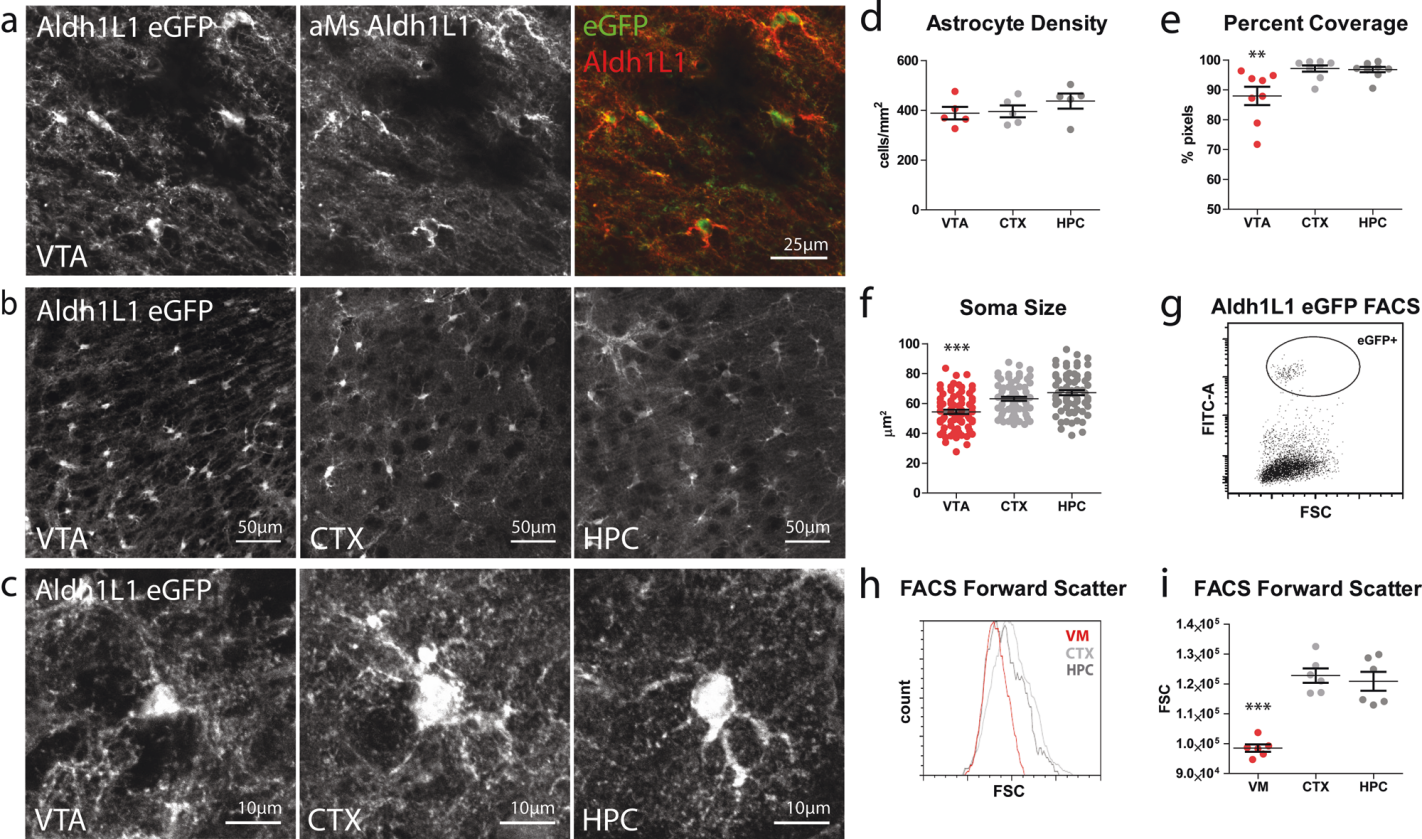
Morphology of ventral midbrain and telencephalic astrocytes. **a** Coronal brain section from a P60 *Aldh1L1-eGFP* mouse immunostained for Aldh1L1 (red). **b** Images taken from P60 *Aldh1L1-eGFP* mouse section showing density and tissue coverage by eGFP+ astrocyte processes in the ventral tegmental area (VTA), cortex (CTX), and hippocampus (HPC). **c** Closeup of individual eGFP+ astrocytes. **d** Quantification of eGFP+ astrocyte density in P60 animals. One-way ANOVA, *F* = 0.6611, *p* = 0.5441, *N* = 5 animals, 1 section per animal, per region. **e** Quantification of percent tissue coverage by eGFP+ astrocyte processes in P60 animals (see Methods for details). One-way ANOVA, *F* = 7.866, *p* = 0.0025; Bonferroni post hoc tests for CTX vs HPC *t* = 0.1441, CTX vs VTA *t* = 3.536, HPC vs VTA *t* = 3.396 (**p < 0.01). *N* = 8–9 sections, 4–5 animals per region. **f** Quantification of eGFP+ astrocyte soma size in P60 animals. One-way ANOVA, *F* = 19.52, *p* < 0.0001; Bonferroni post hoc tests for CTX vs HPC *t* = 1.899, CTX vs VTA *t* = 4.179, HPC vs VTA *t* = 6.078 (***p < 0.0001). *N* = 6–8 sections, 4–6 animals per region. **g** Example scatter plot of one FACS experiment, with forward scatter values on the *x*-axis and FITC channel intensity on the *y*-axis. Circle indicates eGFP+ astrocyte considered in analysis. **h** Forward scatter values from one FACS experiment in which Aldh1L1-eGFP tissue microdissected from ventral midbrain, CTX, and HPC were dissociated into single-cell suspensions and analyzed by flow cytometry. Graph shows aggregate forward scatter values of eGFP+ astrocytes from the three regions. **i** Quantification of average forward scatter values (per animal/experiment) of eGFP+ astrocyte from ventral midbrain, CTX, and HPC. One-way ANOVA, *F* = 31.06, *p* < 0.0001; Bonferroni post hoc tests for CTX vs HPC *t* = 0.5599, CTX vs ventral midbrain *t* = 0.7089, HPC vs ventral midbrain *t* = 6.529 (***p < 0.001). *N* = 6 animals/experiments

Astrocytes exhibit a tiled tissue distribution and blanket the entire CNS with their remarkably complex, finely branched processes that ensheath synapses [22] and make specialized contacts with blood vessels [23]. As such, the morphology of these cells yields important information about the extent of functional interactions between these cells and surrounding structures. To visualize astrocytes, we used the *Aldh1L1-eGFP* line of transgenic mice, which labels all astrocytes as well as their fine processes [24, 25], and imaged astrocytes in the VTA—one of the two dopaminergic nuclei in the ventral midbrain—as well as cortex and hippocampus of 2-month-old mice. eGFP expression is confined to cells that express endogenous Aldh1L1 (Fig. 1a) and does not overlap with cell-type-specific markers for oligodendrocytes, NG2 cells, or neurons (Figure S1a). Although the density of astrocytes was comparable across all three regions (Fig. 1b, d), the percentage of tissue coverage by astrocyte processes was significantly lower in the VTA relative to cortex and hippocampus (Fig. 1c, e; Figure S1b). In addition, VTA astrocytes had significantly smaller somas, as assessed by both confocal image analysis (Fig. 1c, f) and flow cytometry analysis of acutely dissociated Aldh1L1-eGFP+ cells (Fig. 1g, h, i). Together, these results indicate that ventral midbrain astrocytes are distinct from telencephalic astrocytes in their basic morphological properties.

### Ventral midbrain astrocytes are molecularly distinct from telencephalic astrocytes

**Fig. 2:**
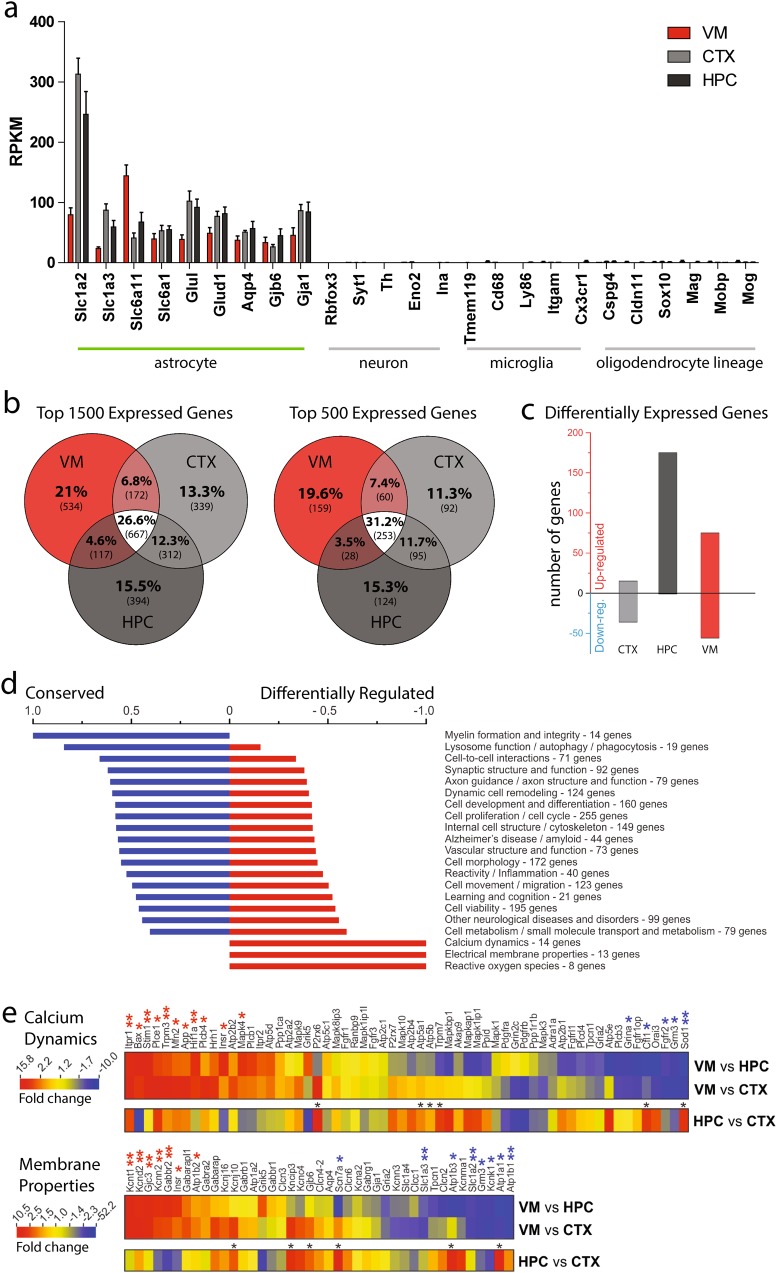
Transcriptomic analysis of ventral midbrain and telencephalic astrocytes. **a** RPKM values of cell-type-specific genes obtained from RNA sequencing of Aldh1L1-eGFP+ astrocyte transcripts (see Methods for details). **b** Degree of overlap among the top 1500 or top 500 expressed genes in each region, using mean RPKM. **c** Number of significantly up- or down-regulated genes in each region (EDGE test *p* < 0.05, mean RPKM >2 and norm. SEM < 0.5 in the more highly expressed region). **d** Degree to which genes in various functional families are conserved or differentially expressed (see Methods for details). **e** Heat maps comparing RPKM fold change in VM vs. HPC or CTX astrocytes for genes related to calcium dynamics (top) or electrical membrane properties (bottom). Asterisks above genes indicate EDGE test *p* values (*p < 0.05, **p < 0.01)

To investigate whether differences in astrocyte morphology are accompanied by differences in other aspects of cell phenotype, we isolated eGFP+ astrocytes from the ventral midbrain, cortex, and hippocampus of adult *Aldh1L1-eGFP* mice via flow cytometry and performed RNA sequencing to examine the astrocyte transcriptome in each of these three regions (Figure S2a, b). In samples from all three brain regions, we detected robust expression of well-known astrocyte-specific genes [25], but not genes specific to other CNS cell populations (Fig. 2a).

To define the extent to which the astrocyte transcriptome varies across these regions, we first compared the amount of overlap in either the top 1500 or top 500 most abundantly expressed genes in each region (Fig. 2b). Roughly 30% of genes were present in gene lists from all three astrocyte populations, and 22% were present in lists from at least 2 astrocyte populations. Strikingly, almost 45% of genes were specific to gene lists of one region. Among genes that were present in lists from only one region, ventral midbrain astrocytes showed the largest number, with roughly 20% of genes not overlapping with cortical and hippocampal astrocyte genes. We next used statistical analysis to identify genes that were significantly up- or down-regulated in each population of astrocytes (Fig. 2c). Hippocampal astrocytes had the greatest number of upregulated genes as compared to the other two regions, whereas ventral midbrain astrocytes had a substantial number of genes that were both up- and down-regulated compared to cortical and hippocampal astrocytes. To determine which molecular pathways are most likely to vary across these astrocyte populations, we compiled lists of the most conserved and the most differentially regulated genes and used Ingenuity Pathway Analysis to determine what functional families these genes fell into (see Methods). Functional families in which gene expression tended to be conserved included myelin formation and integrity, lysosome function, and cell–cell interactions. Unexpectedly, genes related to calcium dynamics, electrical membrane properties, and reactive oxygen species—all core aspects of astrocyte physiology—were more differentially expressed than conserved (Fig. 2d). To expand upon these observations, we generated heat maps showing genes associated with calcium dynamics or electrical membrane properties (Fig. 2e). For both functional families, there were significantly up- and down-regulated genes (16) in ventral midbrain astrocytes as compared to hippocampal or cortical astrocytes. A comparison of hippocampus and cortex revealed fewer differentially expressed genes (6), all of which were upregulated in the hippocampus relative to cortex. Thus, in addition to having significant anatomical differences, ventral midbrain astrocytes also exhibit a unique pattern of gene expression.

### VTA astrocytes are electrophysiologically distinct from telencephalic astrocytes

**Fig. 3:**
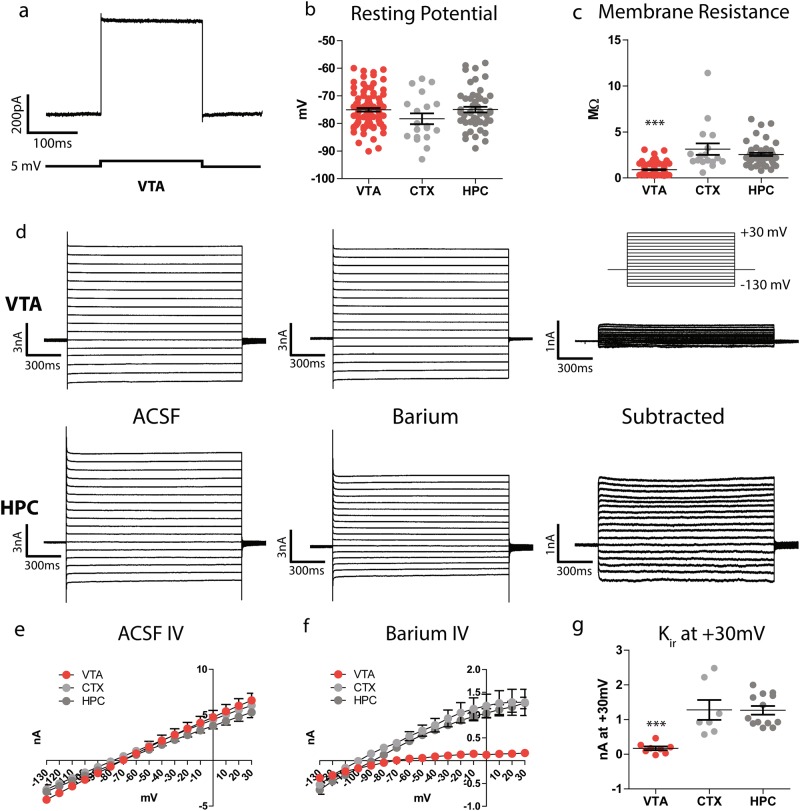
Electrophysiological properties of ventral midbrain and telencephalic astrocytes. **a** Trace from one astrocyte recording in voltage clamp, in which a brief negative voltage step was delivered. **b** Quantification of astrocyte resting potential. One-way ANOVA, *F* = 1.91, *p* = 0.152. *N* = 19 CTX cells (12 animals, 14 slices), 50 HPC cells (20 animals, 22 slices), and 70 VTA cells (28 animals, 32 slices). **c** Quantification of astrocyte membrane resistance. One-way ANOVA, *F* = 49.3, *p* < 0.0001; Bonferroni post hoc tests for CTX vs HPC *t* = 1.764, CTX vs VTA *t* = 5.366, HPC vs VTA *t* = 5.362 (***p < 0.001). *N* = 17 CTX cells (12 animals, 14 slices), 50 HPC cells (20 animals, 22 slices), and 46 VTA cells (22 animals, 23 slices). **d** Example traces of one VTA astrocyte and one HPC astrocyte in voltage clamp, stepped from −130 mV to + 30 mV holding potential in 10 mV increments (voltage step command depicted in inset, top right). Left, current measurements in ACSF; middle, current measurements in 100 µM barium; right, digital subtraction of current measured in barium from current measured in ACSF. **e** Quantification of IV relationship of all cells in ACSF. One-way ANOVA, *F* = 0.09728, *p* = 0.983. *N* = 8 CTX (8 animals, 8 slices), 22 HPC (13 animals, 14 slices), and 37 VTA (16 animals, 19 slices). **f** Quantification of IV relationship of barium-sensitive current for all cells. One-way ANOVA, *F* = 0.1091, p = 0.0035; Bonferroni post hoc tests for CTX vs HPC *t* = 0.7489, CTX vs VTA *t* = 3.397, HPC vs VTA *t* = 2.648. *N* = 7 CTX (5 animals, 7 slices), 13 HPC (10 animals, 13 slices), and 8 VTA (7 animals, 8 slices). **g** Quantification of barium-sensitive current at +30 mV holding potential. One-way ANOVA, *F* = 14.23, *p* < 0.0001; Bonferroni post hoc tests for CTX vs HPC *t* = 0.05326, CTX vs VTA *t* = 4.343, HPC vs VTA *t* = 4.947 (***p < 0.001). N = 7 CTX (5 animals, 7 slices), 13 HPC (10 animals, 13 slices), and 8 VTA (7 animals, 8 slices)

One of the primary functions of astrocytes is to buffer extracellular potassium via the expression of multiple potassium channels [13, 26, 27]. To determine whether the differences in astrocyte gene expression that we observed are associated with differences in the physiological properties of local astrocyte networks, we prepared acute brain sections from *Aldh1L1-eGFP* mice and performed whole-cell patch clamp recordings from individual astrocytes in the VTA, cortex, and hippocampus (Fig. 3a, S3). Astrocyte resting potential was comparable in all three regions and ranged from –60 mV to –90 mV (Fig. 3b). However, membrane resistance was significantly lower in VTA astrocytes (Fig. 3c), suggesting potentially higher levels of potassium leak channels. The inward-rectifying potassium channel K_ir_4.1 plays a dominant role in shaping astrocyte membrane properties in hippocampus [28, 29], so we predicted that VTA astrocytes may have higher levels of functional K_ir_4.1 channels. To test this, we stepped the holding potential of each cell from −130 mV to +30 mV before and after bath application of barium, a blocker of inward-rectifying potassium channels, to quantify the barium-sensitive potassium current. In ACSF, we observed no voltage-gated currents or rectification (Fig. 3d, left traces; Fig. 3e), whereas the voltage–current relationship of the barium-sensitive current shows an inward rectification and reverses at −90 mV (Fig. 3f), indicating that it is a potassium-mediated current. Unexpectedly, VTA astrocytes exhibited very little barium-sensitive potassium current as compared to cortical and hippocampal astrocytes (Fig. 3f, g). Echoing the sequencing results, these findings indicate that ventral midbrain astrocytes differ from telencephalic astrocytes in their basic electrophysiological properties and likely use a distinct set of ion channels to buffer extracellular potassium.

### VTA astrocytes are extensively coupled to oligodendrocytes

Another key feature of astrocyte physiology is the expansive interconnected network they form together via gap junctions [30]. Although gap junction coupling does not appear to impact hippocampal astrocyte membrane properties [31], we nevertheless wondered whether an increase in the extent of astrocyte coupling may be contributing to the low membrane resistance exhibited by VTA astrocytes. To address this possibility, we included biocytin in our recording pipettes and, following recording, fixed and stained slices with streptavidin to visualize cells that are coupled to the recorded cell. To the extent possible, length of recording time and size of the recording pipette were kept constant across cells. Because even minute variation in these and other experimental parameters can affect the spread of biocytin, we restricted our analysis to a field of view contained within the boundary of biocytin diffusion that is identical in size across experiments.

**Fig. 4:**
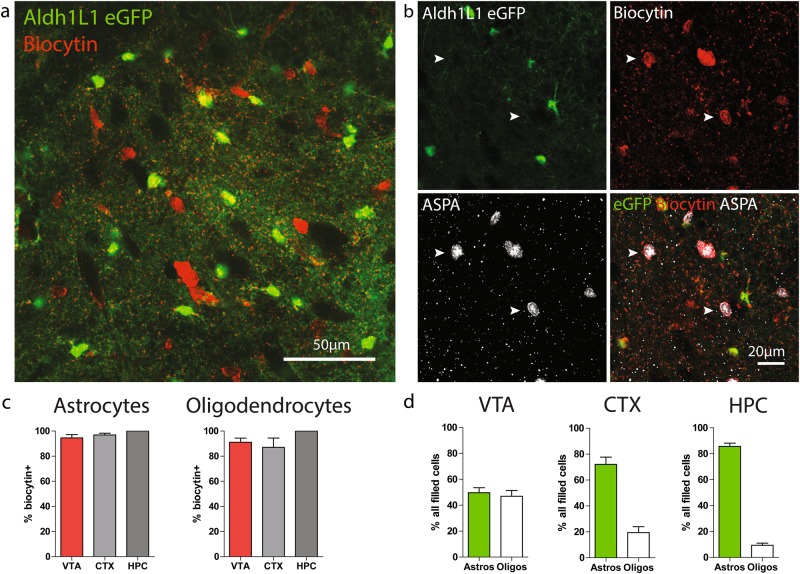
Extensive coupling between astrocytes and oligodendrocytes in the ventral midbrain. **a** Confocal image of a horizontal midbrain section in which one astrocyte was patched and filled with biocytin. Biocytin was visualized by post hoc staining with streptavidin conjugated to Alexa 594. **b** Confocal image of a horizontal midbrain section in which one astrocyte was patched and filled with biocytin, then stained for aspartoacylase (ASPA), a mature oligodendrocyte marker. White arrowheads indicate biocytin+, ASPA+, eGFP− oligodendrocytes. **c** Quantification of the percentage of astrocytes (left) or oligodendrocytes (right) within the biocytin diffusion radius that are biocytin+. *N* = 8 CTX, 5 HPC, and 7 VTA sections. **d** Percentage of network occupied by astrocytes or oligodendrocytes in each region. *N* = 8 CTX, 5 HPC, and 7 VTA sections

Astrocytes across all three regions were extensively coupled to surrounding cells. Surprisingly, in the ventral midbrain, many of the biocytin filled cells were not eGFP+ (Fig. 4a, S4a). Astrocyte–oligodendrocyte gap junction coupling has been reported in other brain regions [32–34], but has been described as infrequent and minor relative to astrocyte–astrocyte coupling [35]. Double staining with aspartoacylase, a marker of mature oligodendrocytes [36], revealed that the vast majority of oligodendrocytes within the biocytin diffusion radius are coupled to astrocytes (Fig. 4b), and oligodendrocytes account for all biocytin+ cells that do not express eGFP (Figure S4b). Coupling between astrocytes and oligodendrocytes was also observed in the hippocampus and cortex (Fig. 4c). However, due to their high density within the midbrain (Figure S4c), oligodendrocytes account for approximately half of this glial network within the VTA, whereas they represent a much smaller fraction of all coupled cells in the cortex and hippocampus (Fig. 4d).

### Ventral midbrain astrocytes are relatively insensitive to norepinephrine but respond to dopamine D2 receptor modulation

Cytosolic elevations in calcium are a prominent feature of astrocytes and play critical roles in many aspects of their biology and interactions with surrounding cells [37]. Thus, we carried out experiments to determine whether ventral midbrain astrocytes share similar patterns of calcium elevations to telencephalic astrocytes. To this end, we injected cre-dependent GCaMP6 [38] in the ventral midbrain and hippocampus of adult *GFAP-cre* mice (Figure S5a) and performed two-photon time-lapse imaging of astrocyte calcium activity in acute slices. GCaMP6 expression was largely restricted to astrocytes, as indicated by overlap of GCaMP6 with the astrocyte marker S100β, but not markers of other cell types (Figure S5b,c). A small number of neurons were also infected (Figure S5d) but could be clearly identified by their distinct morphology and excluded from analysis.

**Fig. 5:**
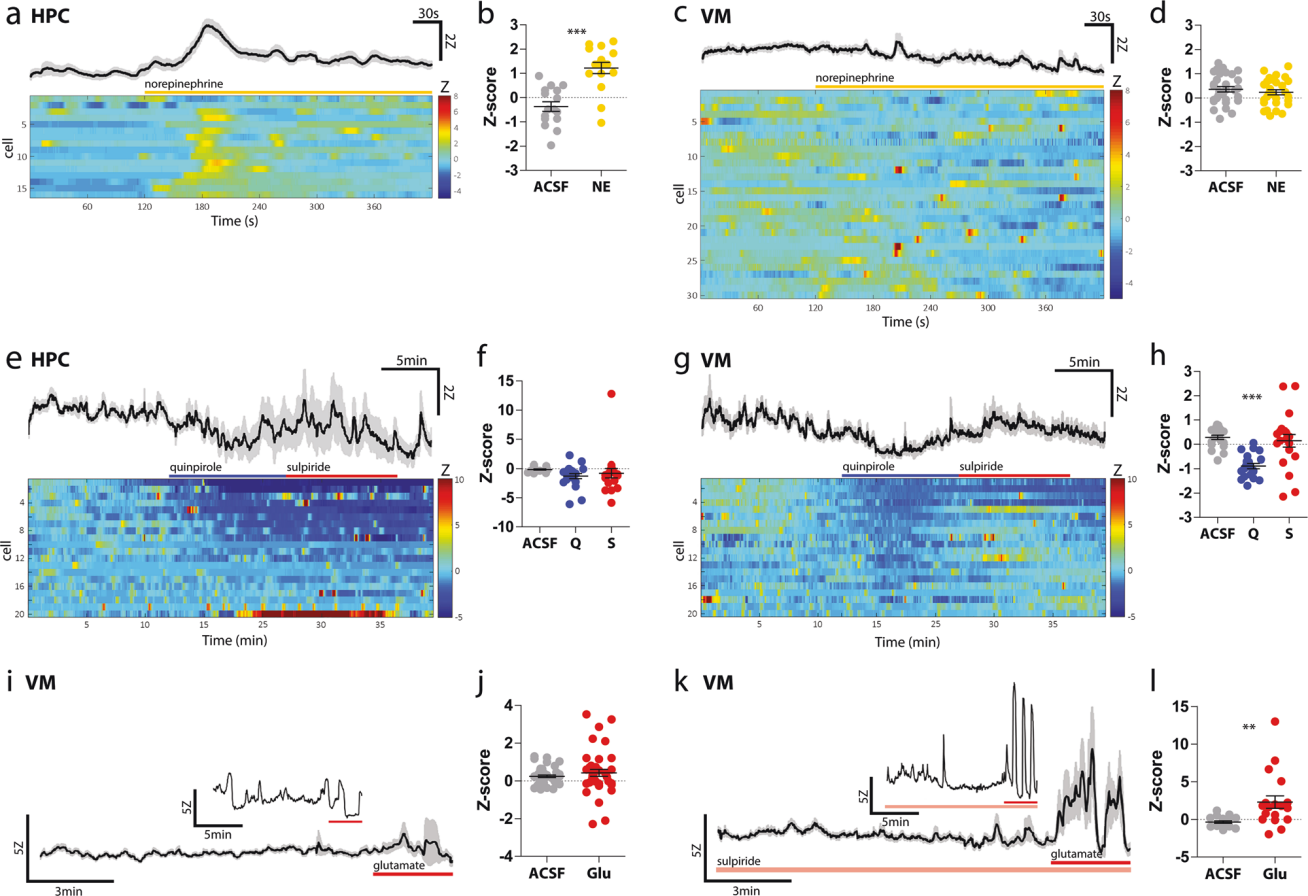
Ventral midbrain and hippocampal astrocyte calcium responses to norepinephrine and D2 receptor modulation. **a** Average (top trace, SEM in gray) and individual (bottom, color raster plot) HPC astrocyte calcium responses to bath application of 40 μM norepinephrine. Raw intensity values were normalized to baseline period as *Z*-scores. **b** Average *Z*-scores of HPC astrocytes 1 min prior to NE (‘ACSF’) and 1 min after onset of NE (‘NE’). Paired *t*-test, *t* = 5.301, *p* < 0.001. *N* = 5 slices, 2 animals. **c** Average and individual ventral midbrain astrocyte calcium responses to bath application of 40 μM norepinephrine. **d** Average *Z*-scores of ventral midbrain astrocytes 1 min prior to NE (‘ACSF’) and 1 min after onset of NE (‘NE’). Paired *t*-test, *t* = 0.866, *p* = 0.3936. *N* = 9 slices, 3 animals. **e** Average and individual hippocampal astrocyte calcium responses to 10 μM quinpirole and 300 nM sulpiride. **f** Average *Z*-scores of HPC astrocytes during 5 min of baseline (‘ACSF’), 5 min of quinpirole (‘Q’), and 5 min of sulpiride (‘S’). One-way ANOVA, *F* = 1.167, *p* = 0.3187. *N* = 6 slices, 2 animals. **g** Average (top) and individual (bottom) ventral midbrain astrocyte calcium responses to 10 μM quinpirole and 300 nM sulpiride. **h** Average *Z*-scores of ventral midbrain astrocytes during 5 min of baseline (‘ACSF’), 5 min of quinpirole (‘Q’), and 5 min of sulpiride (‘S’). One-way ANOVA, *F* = 14.07, *p* < 0.0001. Bonferroni post hoc tests for ACSF vs Q *t* = 4.858, *p* < 0.001; ACSF vs S *t* = 0.5823, *p* > 0.05; Q vs S *t* = 4.276, *p* < 0.001. *N* = 7 slices, 2 animals. **i** Calcium response of an individual ventral midbrain astrocyte (inset) and average response from all imaged ventral midbrain astrocytes to 1 mM glutamate (duration of application indicated by red bar), in the presence of 500 μM lidocaine, 100 μM kyneurinic acid, 100 μM picrotoxin, and 1 μM CGP35348. **j** Average *Z*-scores of ventral midbrain astrocytes 2 min prior to glutamate (‘ACSF’) and 2 min after onset of glutamate (‘Glu’). Paired *t-*test, *t* = 1.001, *p* = 0.3228. *N* = 13 slices, 4 animals. **k** Calcium responses of an individual ventral midbrain astrocyte (inset) and average response from all imaged to 1 mM glutamate (duration of application indicated by red bar), in the presence of 300 nM sulpiride, 500 μM lidocaine, 100 μM kyneurinic acid, 100 μM picrotoxin, and 1 μM CGP35348. **l** Average *Z*-scores of ventral midbrain astrocytes 2 min prior to glutamate (‘ACSF’) and 2 min after onset of glutamate (‘Glu’). Paired *t*-test, *t* = 3.145, *p* = 0.0056. *N* = 6 slices, 2 animals

As a positive control for our readout, we exposed slices to norepinephrine (NE), which has been shown to elicit large calcium elevations in cortical and hippocampal astrocytes [39–41]. Similar to previous reports, NE produced a reliable elevation in the cytosolic calcium levels of hippocampal astrocytes (Fig. 5a, b); unexpectedly, however, NE failed to elicit an obvious calcium response in ventral midbrain astrocytes (Fig. 5c, d). Accordingly, although we detected expression of the NE receptor *Adra1a* in hippocampal and cortical astrocytes using RNAseq, expression of *Adra1a* in ventral midbrain astrocytes did not meet our criterion for an expressed gene (see Methods for definition of expressed genes). Thus, compared to hippocampal astrocytes, ventral midbrain astrocytes are less responsive to NE.

In addition to releasing DA from axonal projections and terminals, DA neurons also exhibit local somato-dendritic DA release [42] where DA binding to Gi-coupled dopamine D2 autoreceptors provides negative feedback to inhibit DA neuron firing [43]. Recent reports suggest that astrocytes can also express dopamine D2 receptors (D2Rs) and that these astrocytic D2Rs can regulate their inflammatory response and DA neuronal survival [7, 44, 45]. However, the molecular mechanisms of this modulation and the impact of D2R activation on astrocyte calcium signaling within dopaminergic nuclei has not been explored. Therefore, we bath applied quinpirole, a D2R/D3R agonist, chased by sulpiride, a D2R/D3R antagonist, and recorded calcium signals in ventral midbrain and hippocampal astrocytes in the presence of blockers of electrical and synaptic activity. Overall, quinpirole produced a decrease in cytosolic calcium in both ventral midbrain and hippocampus astrocytes (Fig. 5e, g), but responses were more consistent in the ventral midbrain (Fig. 5f, h). Sulpiride was only able to reverse this decrease in a subset of ventral midbrain astrocytes (Fig. 5g).

In neurons, DA plays a modulatory role by enhancing or suppressing the effect of signaling from other transmitters such as glutamate. To investigate whether DA signaling can play a similar modulatory role in astrocytes, we bath applied glutamate in the presence or absence of the D2R/D3R antagonist sulpiride. For this analysis, we focused on ventral midbrain astrocytes, which displayed the most reliable responses to quinpirole (Fig. 5f, h) and should be exposed to greater levels of tonic DA signaling in vivo [46]. Collectively, ventral midbrain astrocytes did not display a consistent response to bath application of glutamate alone (Fig. 5i, j; Figure S5e). However, when D2Rs were blocked by sulpiride, ventral midbrain astrocyte calcium responses to glutamate were greatly enhanced (Fig. 5k, l; Figure S5f). These findings suggest that DA signaling can play a key role in shaping ventral midbrain astrocyte calcium activity.

## Discussion

The first hints of astrocyte heterogeneity emerged from Cajal’s pioneering morphological studies [14], but received little attention until recent demonstrations that spatially restricted pools of astrocyte progenitors give rise to distinct populations of mature astrocytes capable of region-specific modulation of neuronal circuit formation [47]. To date, only a handful of studies [32, 48, 49] have directly compared the functional properties of mature astrocytes in different brain regions, none of which have included the ventral midbrain. Thus, our study constitutes the first functional and molecular characterization of astrocytes within the ventral midbrain, a brain region that is critical for reward-related behaviors and particularly sensitive to environmental stressors like hypoxia and neuroinflammation [50, 51]. Furthermore, by performing parallel experiments in telencephalic astrocytes, these data provide key contributions to our nascent understanding of functional astrocyte heterogeneity in the mature CNS.

The numerous functional and molecular differences we observed provide clues as to how ventral midbrain astrocytes may be differentially shaping the midbrain. For example, the lower percentage of tissue coverage by astrocytes in the midbrain may reflect reduced synapse ensheathment, which can affect neurotransmitter uptake and the degree of transmitter spillover [52]. This possibility is particularly relevant in the context of DA neuron activity, as an increase in transmitter spillover can lead to persistent activation of extrasynaptic *N*-methyl-d-aspartate receptors that contribute to excitotoxicity [53]. In addition, astrocyte morphology can be modified by stimuli that trigger plasticity in DA neurons [54]. Thus, a reduction in astrocytic synapse coverage may be a contributing factor to the selective vulnerability of DA neurons to degeneration. It is worth noting that, due to the limitations of two-dimensional confocal imaging, other subtle but functionally important differences in fine astrocyte process branching may have been missed, thus meriting follow-up studies combining sparse labeling with higher-resolution three-dimensional analysis of astrocyte morphology [54].

In addition to distinct morphological attributes, ventral midbrain astrocytes exhibited prominent differences in gene expression compared to telencephalic astrocytes, adding to previous studies that observed notable differences in astrocyte gene expression across different brain regions [48, 49]. Many of the significantly upregulated and downregulated genes in ventral midbrain astrocytes are involved in calcium signaling and electrical membrane properties, two aspects of astrocyte physiology that have been linked to some of their most basic functions, such as the release of gliotransmitters and ion buffering. Notably, mRNA transcripts for both astrocytic glutamate transporters (Slc1a2/GLT1 and Slc1a3/GLAST) were downregulated in the ventral midbrain. The lower levels of glutamate transporter expression by ventral midbrain astrocytes coincides with a lower density of excitatory presynaptic terminals (Figure S6), which suggests that ventral midbrain astrocytes may be adapting their molecular profile to reflect the demands of the local milieu.

The importance of potassium buffering by astrocytes can be appreciated in contexts where astrocyte potassium channels are compromised [55, 56]. In the striatum, for example, targeted reduction of K_ir_4.1 in astrocytes significantly reduces extracellular potassium uptake [56]. The low level of K_ir_-mediated current detected in ventral midbrain astrocytes may indicate a reduced capacity for ventral midbrain astrocytes to buffer potassium, which could shape the ability of DA neurons to maintain and/or shut off burst firing [57]. On the other hand, several non-K_ir_ potassium channels were enriched in ventral midbrain astrocytes as compared to telencephalic astrocytes, including the calcium-activated potassium channels KCNT1 and KCNN2. In future studies, specific targeting of multiple (and non-canonical) astrocyte-enriched potassium channels will be required to elucidate the interplay between astrocyte potassium buffering and DA neuron excitability.

The extensive gap junction network formed by astrocytes is a unique feature of these cells and appears to be important for metabolic trafficking [58, 59] and the redistribution of ions [60, 61]. In contrast to the previously held idea that astrocyte–oligodendrocyte coupling is an infrequent event [35], we found that essentially all oligodendrocytes within the diffusion radius are coupled to astrocytes and make up nearly half the network of coupled cells in the ventral midbrain. Deletion of one astrocyte connexin and one oligodendrocyte connexin results in a loss of astrocytes [33], suggesting that astrocyte–oligodendrocyte coupling is important for astrocyte survival. Given the high proportion of oligodendrocytes in the ventral midbrain glial syncytium, ventral midbrain astrocytes may be especially reliant on neighboring oligodendrocytes (and vice versa) and, therefore, more susceptible to dysfunction or death if this structural coupling is compromised. A recent finding of great relevance is the role of oligodendrocytes in providing metabolic support to axons [62–64]. Their extensive coupling with astrocytes raises the possibility that oligodendrocyte-derived metabolic substrates may also be accessible to cell bodies and other neuronal compartments contacted by astrocyte processes. Given the tonic firing activity of DA neurons, the large number of oligodendrocytes in the ventral midbrain glial network, and the potential involvement of neural metabolic deficits in neurodegeneration [65], an intriguing question emerging from these findings is whether/how oligodendrocytes might be involved in maintaining the metabolic demands of DA neurons.

Previous work has suggested that deletion of astrocyte D2Rs enhances the release of astrocytic inflammatory factors and aggravates DA neuron injury from the neurotoxin MPTP (1-methyl-4-phenyl-1,2,3,6-tetrahydropyridine), although the mechanism by which this occurs was not explored [7]. Given the existing literature linking rises in astrocyte calcium to increased release of various signaling factors, we predicted that D2R activation would reduce astrocyte calcium activity. Indeed, we found that the D2R agonist quinpirole produced a consistent decrease in astrocyte cytosolic calcium, particularly in ventral midbrain astrocytes. Notably, this decrease was only reversed by the D2R antagonist sulpiride in a subset of astrocytes, indicating that in some cells, D2R-mediated depression of cytosolic calcium becomes at some point independent of ongoing D2R activation. The heterogeneous responses to sulpiride may reflect individual differences in the expression of other calcium regulators which synergize with D2R signaling (e.g., voltage-gated calcium channels that are inactivated by calcium [66]) and suggests that single-cell analysis of gene expression is merited to tease out functional differences of individual astrocytes within the ventral midbrain.

In additional to the consistent effect of quinpirole on baseline calcium levels, we observed a pronounced enhancement of astrocyte calcium responses to glutamate when we blocked D2Rs with sulpiride, possibly reflecting a tonic level of D2R activity in the ventral midbrain that tempers astrocyte calcium responses to stimuli. Although we cannot rule out the possibility that these effects are mediated by the D2Rs expressed in DA neurons, the presence of lidocaine and synaptic blockers in these experiments suggests that they are at least independent of changes in neuronal firing activity.

The responsiveness of ventral midbrain astrocytes to D2R modulation also carries important implications in the context of drug-seeking behavior. Although there is no question that D2 autoreceptor activity critically regulates DA neuron activity, a downregulation of astrocytic D2Rs by chronic drug exposure may also be partly responsible for drug-induced plasticity in the ventral midbrain. Similarly, enhancing or dampening astrocytic D2Rs may partly account for the effects of D2R-targeted drugs or genetic manipulation of D2R expession on drug-seeking behavior [67–70]. With the development of new, selective astrocyte mouse lines [71], future studies will be able to define the relative contribution of astrocytic and DA neuron D2Rs to drug-related plasticity and behaviors.

A fundamental anatomical difference between the ventral midbrain and telencephalic regions is the extensive mingling of myelinated axons and cell bodies/dendrites, such that the ventral midbrain cannot be accurately classified as either a white matter or a gray matter region. Although some of the properties we described here (e.g., enhanced responsiveness to D2R modulation) is likely related to the presence of dopamine neurons, others may result from balancing the demands of different neuronal compartments. The sheer amount of space occupied by myelinated axons and oligodendrocytes within the ventral midbrain may explain the reduced tissue coverage by astrocyte processes. As a result, the ability of astrocytes to access neuronal structures may be impeded, therefore requiring astrocytes and oligodendrocytes to work in concert to fulfill homeostatic tissue functions. Aside from potentially contributing to the metabolic demands of neurons, oligodendrocytes might help buffer extracellular potassium, since they also express K_ir_ channels [72]. Addressing these possibilities will require complimentary studies of oligodendrocyte properties in different brain regions, particularly as they relate to astrocyte properties and functions. It is clear, however, that the classic delineation of white matter fibrous astrocytes and gray matter protoplasmic astrocytes is insufficient to capture the complexity of astrocyte phenotypes present throughout the brain.

In conclusion, our data represent the first comprehensive description of ventral midbrain astrocytes and further define the extent of regional astrocyte heterogeneity in the adult brain. The unique features of ventral midbrain astrocytes—including their differential expression of potassium channels, extent of coupling to oligodendrocytes, and modulation by D2Rs—may be relevant for our understanding of how the ventral midbrain adapts in pathological contexts, such as substance abuse and Parkinson’s disease. Future studies on neuron–glial interactions in this region will need to consider the region-specific phenotype of ventral midbrain astrocytes when evaluating their contribution to DA neuron function, plasticity, and survival.

## Electronic supplementary material

Figure S1

Figure S2

Figure S3

Figure S4

Figure S5

Figure S6

Table S1

Table S2
