# Supplementary material for: Ventral midbrain astrocytes display unique physiological features and sensitivity to dopamine D2 receptor signaling
Source: Neuropsychopharmacology. 2018 Jul 13;44(2):344–55. doi: 10.1038/s41386-018-0151-4 (PMC6300565; doi:10.1038/s41386-018-0151-4)
Supplement: Supplementary file 7 — Table S1 [file 41386_2018_151_MOESM7_ESM.docx]

**Supplementary Table 1: Primary Antibodies**

| **Name** | **Species** | **Company** | **Catalog** | **Dilution** |
| --- | --- | --- | --- | --- |
| Aldh1l1 | Ms | Millipore | MABN495 | 1:500 |
| S100β | Rb | Millipore | 04-1054 | 1:500 |
| Tyrosine Hydroxylase (TH) | Ms | Millipore | MAB318 | 1:1000 |
| Aspartoacylase (ASPA) | Rb | Genetex |  | 1:2000 |
| NG2 | GP | Courtesy Dr. Dwight Bergles | N/A | 1:50 |
| CNP | Rb | Cell Signaling | 5664 | 1:100 |
| Iba1 | Rb | Wako | 019-19741 | 1:500 |
| NeuN | Ms | Millipore | MAB377 | 1:1000 |
| Aquaporin 4 (Aqp4) | Rb | Santa Cruz | Sc-20812 | 1:500 |
| Alexa Fluor 594 Streptavidin | N/A | Jackson ImmunoResearch | 016-580-084 | 1:500 |
| VGluT2 | GP | Frontier Institute | VGluT2-GP-Af810 | 1:500 |
| VGluT1 | GP | Millipore | AB5905 | 1:500 |
| VGluT3 | GP | Synaptic Systems | 135 204 | 1:500 |
| VGluT3 | Rb | Synaptic Systems | 135 203 | 1:500 |
