# Supplementary material for: Ventral midbrain astrocytes display unique physiological features and sensitivity to dopamine D2 receptor signaling
Source: Neuropsychopharmacology. 2018 Jul 13;44(2):344–55. doi: 10.1038/s41386-018-0151-4 (PMC6300565; doi:10.1038/s41386-018-0151-4)
Supplement: Supplementary file 8 — Table S2 [file 41386_2018_151_MOESM8_ESM.pdf]

	VM - Means	SEM	SEM/Mean
Gm10800	8950.800875	3815.079	0.426228
Gm26870	2920.068893	1234.344	0.422711
Gm21738	2604.026582	1065.157	0.409042
Malat1	2141.974938	422.4085	0.197205
Gm10801	1611.142217	686.2774	0.425957
mt-Co1	938.1960715	139.1066	0.14827
mt-Nd4	924.3118279	169.6937	0.183589
mt-Cytb	745.801716	117.6571	0.157759
mt-Nd1	690.6017828	58.35416	0.084498
mt-Co2	490.8186578	62.27044	0.126871
mt-Atp6	470.0121692	74.24531	0.157965
mt-Nd5	439.6182004	55.37792	0.125968
mt-Nd2	329.5720914	85.71234	0.260072
Ndrg2	305.8438252	40.02246	0.130859
mt-Nd4l	279.7879479	25.26596	0.090304
mt-Co3	239.0129274	88.96851	0.372233
Sparcl1	218.3495837	33.27333	0.152386
mt-Atp8	200.3285947	59.72824	0.298151
Atp1b2	149.7959917	26.59868	0.177566
Slc6a11	144.3096461	17.80757	0.123398
Cst3	136.459157	53.06689	0.388885
Gm10721	108.5561244	50.92877	0.469147
Ntsr2	90.69555274	24.03959	0.265058
Atp1a2	90.62706668	16.53665	0.182469
mt-Nd6	88.25427272	40.07246	0.454057
Mt2	84.18285077	28.07107	0.333454
Slc1a2	79.53513884	11.63019	0.146227
Ckb	77.27314262	19.14541	0.247763
Cd81	76.38696207	13.49054	0.176608
Sparc	70.47259629	14.40915	0.204465
Cpe	68.35222532	18.86766	0.276036
Lars2	67.54867169	13.85653	0.205134
Aldoc	66.21455499	7.596099	0.114719
Apoe	65.1984476	17.80335	0.273064
Scd2	63.07980318	15.39306	0.244025
Gstm1	60.17300621	12.13391	0.20165
Itih3	58.75454631	14.6445	0.249249
Tecr	56.31435324	10.30865	0.183055
Ppap2b	51.39812499	11.22522	0.218397
Ptprz1	50.89462177	6.449412	0.126721
Gm3764	50.34304856	8.544644	0.169728
Glud1	49.10828094	9.120549	0.185723
Bcan	48.3134345	8.648216	0.179002
Snrnp70	47.7834649	11.91631	0.249381
Acsl3	47.24000759	7.612725	0.16115
Gja1	45.75529788	12.12244	0.264941

Gm21292	43.54036849	13.98761	0.321256
Slc4a4	43.37072588	8.927463	0.205841
Hnrnpa2b1	42.24314676	13.16089	0.311551
mt-Nd3	41.85778201	11.9006	0.28431
Mid1	40.04589124	17.26185	0.431052
Gpr37l1	39.92556038	16.59305	0.4156
Slc6a1	39.41991829	8.780026	0.222731
Glul	38.79249423	7.642886	0.19702
Aqp4	37.48386663	6.850747	0.182765
Slc27a1	36.55149642	6.849037	0.18738
Ddx17	36.43492399	6.50095	0.178426
Prnp	36.43202721	16.41555	0.45058
Tmem47	34.18536582	9.97479	0.291785
Agt	33.8884542	16.22233	0.478698
Gjb6	33.57805586	8.634765	0.257155
Atp5b	33.30354641	5.108802	0.153401
Gm21820	32.91219808	13.10113	0.398063
Nrbp2	32.7082042	8.489226	0.259544
S1pr1	32.19236905	6.771957	0.210359
Gm26818	32.09562411	7.127471	0.22207
Clu	31.9564967	6.400279	0.200281
Ttyh1	31.71790443	1.874549	0.059101
Dbi	31.47116608	10.46638	0.33257
Appl2	31.27113969	9.07367	0.290161
Csrp1	30.9995247	11.54755	0.372507
Srsf5	30.92636754	8.218905	0.265757
Sepp1	30.83223078	4.9988	0.162129
Nnat	27.7333371	13.33917	0.48098
Trim9	27.46961238	2.932824	0.106766
Psap	27.21712854	9.689425	0.356005
Acsbg1	27.16075067	9.265151	0.341123
Gpm6a	26.70650719	3.174088	0.118851
Gm10715	26.23474337	12.96509	0.494195
Ywhae	26.21919903	3.597346	0.137203
Fabp7	25.96899514	4.029741	0.155175
Atp13a4	24.80659319	6.678654	0.269229
Ptn	24.72174774	3.65225	0.147734
Ntrk2	24.37211248	2.100712	0.086193
Leng8	24.36253653	3.634145	0.149169
Nwd1	24.23776462	6.570972	0.271105
Cwc22	24.00055357	10.51204	0.437992
Scg3	23.96733989	6.308653	0.263219
Cat	23.88138491	9.595786	0.40181
Ncam1	23.79716789	1.124068	0.047235
Slc1a3	23.73334857	2.741683	0.11552
Macf1	23.3468277	3.8811	0.166237
Gm20594	23.29467046	8.067801	0.346337

Gdpd2	22.72255116	5.761347	0.253552
Ldhb	22.48775426	4.71768	0.209789
Ptch1	22.41102012	7.346943	0.327827
Serpine2	22.23841572	4.609825	0.207291
Kcnj10	22.00009623	4.870612	0.22139
Tril	21.71144637	10.34472	0.476464
Ubc	21.21826258	8.958785	0.42222
Car2	20.93916147	6.029496	0.287953
Gas5	20.92902629	4.708696	0.224984
Atp2a2	20.83955631	6.655477	0.319368
Fads1	20.80746673	4.221084	0.202864
Ncan	20.55531192	3.830715	0.186361
Psd2	19.85524099	6.78091	0.341517
Tpp1	19.79662032	6.637817	0.335301
Lamp1	19.72157458	5.06932	0.257044
Lcat	19.56155961	6.648184	0.33986
Mat2a	19.17303013	6.503753	0.339214
Slc39a12	19.08769994	5.219202	0.273433
Srrm2	18.858092	3.755819	0.199162
Acsl6	18.83926073	3.904365	0.207246
Rnasek	18.4812458	6.673781	0.361111
Fam107a	18.46670175	6.083954	0.329455
Son	18.43655455	4.29779	0.233112
Luzp2	18.36806519	4.57894	0.249288
Nr1d1	17.9474333	4.404021	0.245384
Gpld1	17.7360766	5.199379	0.293153
Hsp90b1	17.68450174	6.264732	0.35425
Slc25a4	17.61714568	5.034118	0.285751
Dtna	17.50481966	5.596063	0.319687
Mtss1l	17.4153044	8.624363	0.495217
Sat1	17.40061317	4.623856	0.265729
6-Mar	17.22029301	3.559663	0.206713
Ogt	17.11343148	4.594314	0.268462
Elovl5	16.99456822	4.143453	0.24381
Ednrb	16.9815467	4.033016	0.237494
Ddx5	16.79786453	5.589835	0.332771
Tuba1a	16.77047747	5.286841	0.315247
Fxyd1	16.75367377	6.465462	0.385913
Ddhd1	16.74608308	3.401975	0.20315
Igsf11	16.4744848	7.165195	0.434927
Kidins220	16.38921746	2.994223	0.182695
Dazap2	16.37121712	7.953951	0.48585
Actb	16.23494528	5.125073	0.315682
Hsp90ab1	16.16959445	4.219756	0.260969
Ahcyl1	16.09802594	4.557519	0.28311
Neat1	16.09139503	6.684183	0.415389
Fads2	16.00668047	2.631698	0.164412

Trpm3	15.98945772	2.970451	0.185776
Tst	15.97804524	4.608585	0.288432
Hspd1	15.83852433	6.316062	0.398778
Itm2b	15.7214986	4.27815	0.272121
Sash1	15.69828686	4.901637	0.31224
Itm2c	15.67378257	7.469244	0.476544
Hepacam	15.49370016	3.775858	0.243703
Il18	15.39827839	5.64106	0.366344
Cnp	15.35930669	7.115262	0.463254
Pnn	15.15134323	2.076607	0.137058
Mrgprx1	14.89854699	5.274274	0.354013
Ctsl	14.79530989	6.60004	0.44609
Gm10557	14.73047544	6.987434	0.474352
Lrig1	14.71001585	2.504047	0.170227
Kif1a	14.52146599	2.723718	0.187565
Camk2g	14.4391867	3.164844	0.219184
Clip3	14.4166844	5.073611	0.351926
2310022B(	14.20469856	4.358895	0.306863
Gdi1	14.1052244	2.754684	0.195295
Phka1	14.0546958	2.211152	0.157325
Spon1	13.96303545	1.802733	0.129108
Daam2	13.92333661	5.463105	0.39237
5031439G(	13.91322586	2.003838	0.144024
Adcyap1r1	13.9107764	3.485088	0.250532
Luc7l3	13.81005673	3.779143	0.273651
Rac1	13.76031887	5.717657	0.415518
Arhgap5	13.74971985	6.681032	0.485903
Asrgl1	13.71877135	6.254317	0.455895
Atp5a1	13.54084871	4.150469	0.306515
Cyp2j9	13.31619635	5.283957	0.396807
Slc41a1	13.23866171	4.637253	0.350281
C130071C(	13.20358222	2.948584	0.223317
Prrc2c	13.10502259	1.939137	0.147969
Pla2g7	13.06593902	2.519324	0.192816
F3	13.01437967	4.539409	0.348799
Vegfa	12.92545246	3.881398	0.300291
Cyp2j6	12.89995199	2.893389	0.224295
Gm14410	12.86292369	4.467132	0.347287
Tspan3	12.72108325	4.380138	0.344321
Clk1	12.68085385	2.93893	0.231761
Dctn4	12.6618315	6.178418	0.487956
Sod1	12.65124308	5.79621	0.458153
Paics	12.61027778	4.707677	0.373321
Nxf1	12.52277917	5.843411	0.466622
Gstm5	12.40868008	5.193885	0.418569
Pmp22	12.30367937	4.736908	0.384999
Gpd2	12.26922314	2.321598	0.189221

Prdx6	12.18286937	1.716039	0.140857
Srsf3	12.08979141	2.810443	0.232464
4833420G:	12.05259569	5.505443	0.456785
Slc7a10	11.90617624	3.129838	0.262875
Gm21811	11.84079536	3.172239	0.267908
Gpm6b	11.79403621	2.654157	0.225042
Rbmx	11.73442978	4.01796	0.342408
Slc2a1	11.64119415	4.13067	0.354832
Fermt2	11.50744532	4.240753	0.368523
Gm21847	11.47980736	3.20589	0.279263
Gm21882	11.44921428	5.255668	0.459042
Rab7	11.3707625	2.51415	0.221107
Hnrnpk	11.29280927	1.574761	0.139448
Timp3	11.27980835	3.103734	0.275158
Arhgef12	11.23643478	2.240398	0.199387
Fgfr3	11.08778057	4.101264	0.36989
Pcdh9	11.08452726	1.874796	0.169136
Trak1	11.04265679	2.136338	0.193462
Eif4a2	10.92022847	2.78336	0.254881
Ewsr1	10.85268588	4.143749	0.381818
Ctsd	10.7884817	2.898408	0.268658
Aplp2	10.77847542	5.211897	0.483547
Rtn3	10.76307009	3.289275	0.305607
Mpp6	10.7185238	5.279942	0.4926
Cetn3	10.5227015	5.122428	0.486798
Mrfap1	10.4210399	3.499581	0.335819
Gapdh	10.36657708	4.918908	0.474497
Gjc3	10.3290897	2.3438	0.226913
Padi2	10.29340631	3.800032	0.369171
Spire1	10.26057515	3.462756	0.337482
7-Sep	10.23025864	3.80293	0.371733
Clasp2	10.13554329	0.981698	0.096857
Calm1	10.0802548	2.231232	0.221347
Gpam	10.06701232	3.813205	0.378782
Vezf1	10.00230906	2.391514	0.239096
Lsamp	9.997401744	1.34512	0.134547
Spry2	9.985689768	3.249305	0.325396
Sema6d	9.97109111	1.877161	0.18826
Paxbp1	9.906290155	2.75241	0.277845
Canx	9.89192342	1.969497	0.199102
Clk4	9.859617007	1.130748	0.114685
Slc14a1	9.810238665	2.992322	0.30502
Gabbr2	9.734012981	3.893173	0.399956
Cnbp	9.719303576	3.387625	0.348546
Smarcc2	9.669398695	2.412723	0.249522
Pitpnc1	9.656757132	2.302189	0.238402
Lgi1	9.593743462	1.810537	0.188721

4930402H:	9.576935712	2.009252	0.209801
Gabbr1	9.551474378	2.510127	0.2628
Sox9	9.547619083	2.235288	0.23412
Fam213a	9.543551557	4.3467	0.455459
Slc25a23	9.531589404	2.684386	0.28163
Sdha	9.402057905	3.693585	0.392849
Tnik	9.396840223	2.576727	0.274212
Etnppl	9.391811906	2.21821	0.236186
Ccdc88a	9.360819529	0.675573	0.07217
Pygm	9.356469304	4.15866	0.444469
Gm26825	9.315823522	2.488579	0.267135
Cyp51	9.295689576	3.801696	0.408974
Adrbk2	9.268739977	2.044629	0.220594
Pygb	9.230364877	3.704555	0.401344
Nckap1	9.174243694	1.381585	0.150594
Rbm12b1	9.144804064	3.943416	0.431219
Spag9	9.138906447	2.695173	0.294912
Chchd10	9.112916019	2.646716	0.290436
Zfp871	9.095026327	1.724303	0.189587
Laptm4a	9.070833791	2.14604	0.236587
Pbxip1	9.029393484	1.926608	0.213371
Kcnn2	8.974229507	1.337802	0.149071
Rab21	8.969847789	4.449657	0.496068
Cox8a	8.961279555	3.864949	0.431294
Trps1	8.949744807	1.433726	0.160197
Fmn2	8.826732156	2.930356	0.331986
Cd164	8.81907058	2.786379	0.315949
Grina	8.793702902	3.856154	0.438513
B2m	8.780773052	1.58021	0.179963
Paqr6	8.77405454	1.81241	0.206565
Rps29	8.7451656	2.637331	0.301576
Prpf38b	8.726170439	4.29229	0.491887
Fam21	8.682696598	3.342428	0.384953
Ppp1r3c	8.678933999	2.814701	0.324314
Psenen	8.664721194	3.211167	0.370602
Mlc1	8.647657105	3.492876	0.40391
Gabrb1	8.609829159	2.562635	0.297641
Chtop	8.601674375	2.637207	0.306592
Tprkb	8.600963046	3.844102	0.446939
Syt11	8.557925834	2.321685	0.271291
Zcchc7	8.550733031	2.111913	0.246986
Folh1	8.522322766	2.209946	0.259313
Mettl7a1	8.509598338	2.130884	0.250409
Lrp1	8.500733446	1.974792	0.232308
Insig1	8.480329133	2.449161	0.288805
Rbbp9	8.446212924	3.75071	0.44407
Actg1	8.4355053	3.092804	0.366641

Sox2ot	8.424381014	2.399676	0.284849
Tmem229a	8.390177609	3.113067	0.371037
Atl2	8.376378568	2.786128	0.332617
Astn1	8.337529454	2.601589	0.312034
Nrxn1	8.301569304	1.179873	0.142126
Mfge8	8.292263306	2.642576	0.31868
Paqr8	8.255062625	2.536097	0.307217
Gatad2b	8.252061786	3.781128	0.458204
Cyp2d22	8.247883443	3.428553	0.415689
Pdk2	8.200771397	2.02766	0.247252
Zfp825	8.185831309	2.370344	0.289567
Chd9	8.1733075	2.386758	0.292019
Jam2	8.14659842	2.349427	0.288394
Lamp2	8.143568131	2.565077	0.314982
Srsf1	8.123772016	1.512765	0.186215
Srek1	8.102756373	3.983506	0.491624
Wasf3	8.093046709	2.406876	0.297401
Kif1b	8.09077536	0.950873	0.117526
Tjp1	8.08095935	2.779151	0.343914
Arrb2	8.055672403	3.775381	0.468661
Slc1a4	8.036293757	3.345937	0.416353
Tbc1d19	8.022263484	3.61873	0.451086
Hnrnph1	8.016724975	2.524233	0.314871
Fry	8.008917993	1.377315	0.171973
Uba1	7.987862765	2.613218	0.327149
Bclaf1	7.942412348	0.968479	0.121938
Ctnnd2	7.933085656	2.045794	0.257881
Zfp207	7.907009844	2.284623	0.288936
Gria2	7.895162655	1.721395	0.218032
Igfbp5	7.892667943	2.879994	0.364895
Slc6a9	7.887425565	2.828739	0.358639
Mical2	7.883848916	0.630344	0.079954
Rps25	7.880347883	3.164669	0.40159
Scd1	7.816332149	2.265115	0.289793
Ywhaz	7.805367377	2.027264	0.259727
Myo6	7.75061342	2.396777	0.309237
Snapin	7.737669282	3.111047	0.402065
Ntrk3	7.706671405	2.392281	0.310417
Slc7a2	7.706535478	2.340149	0.303658
Rps17	7.680409496	3.369416	0.438703
AI464131	7.672068267	3.549526	0.462656
Mmd2	7.660719542	2.358124	0.30782
Rsrp1	7.656983062	3.665783	0.47875
Cdk2ap1	7.633099557	3.530855	0.462572
Trmt112	7.609660023	3.715295	0.488234
Pigs	7.603470148	2.384157	0.313562
Zbed6	7.581492861	2.565322	0.338366

Sfxn5	7.559478988	1.741647	0.230393
Trib2	7.555045718	2.813345	0.37238
Rnd2	7.549563258	1.996759	0.264487
2610203C2	7.535478908	2.213144	0.293697
Serinc1	7.519515357	1.77683	0.236296
Enho	7.513434542	3.047411	0.405595
Sorl1	7.507027462	2.564295	0.341586
Apc	7.496544804	2.021512	0.269659
Stx16	7.48792702	2.894098	0.386502
Trp53bp2	7.484080361	2.516778	0.336284
Tubb2a	7.483041129	2.142182	0.286272
Kcnd2	7.481781402	1.866384	0.249457
Kcnt1	7.475913603	1.957342	0.26182
Pfkp	7.392514253	2.920088	0.395006
Jmjd1c	7.374144557	1.58219	0.214559
Ncoa4	7.340908103	3.260728	0.444186
Npm1	7.339688734	2.873547	0.391508
Fam214a	7.31220258	1.267842	0.173387
Cs	7.269443483	2.238643	0.307952
Rps9	7.263763621	2.250537	0.309831
Rps6kb2	7.243058088	3.529158	0.487247
Epc1	7.205200904	2.22127	0.308287
Gnao1	7.193456061	2.597761	0.361128
Ocrl	7.176353614	2.15585	0.30041
Peg3	7.135748207	1.562372	0.21895
Ncoa6	7.134745679	2.779564	0.389581
Cadm2	7.113839234	1.555581	0.21867
Slc38a3	7.112447101	1.831651	0.257528
Arglu1	7.091199241	2.748302	0.387565
Eps8	7.082970805	2.658759	0.375373
Cyp7b1	7.078604157	2.576001	0.363914
Atxn2	7.060770365	3.026073	0.428575
Gm20918	7.057778135	2.817069	0.399144
Farp1	7.034195647	1.916532	0.272459
Zkscan3	7.025033468	2.558212	0.364157
Polr2k	7.015927736	3.502518	0.499224
Etv5	6.974009113	2.48039	0.355662
Astx5	6.966082053	2.428426	0.348607
Aplp1	6.94878108	3.192097	0.459375
4930505O1	6.920194604	2.826457	0.408436
Eps15	6.911944695	1.689127	0.244378
Slc25a5	6.878786036	2.680604	0.389691
Fam184a	6.872037672	2.87161	0.417869
Zcchc24	6.844361389	2.379704	0.347688
Arhgef4	6.813985113	1.903367	0.279332
Klf7	6.806799609	2.973604	0.436858
Prkci	6.805410414	3.154678	0.463554

Gm21092	6.798206798	3.264873	0.480255
Eif1	6.786920593	2.31073	0.340468
Serinc3	6.780478602	2.029026	0.299245
Arhgap21	6.771545986	1.750158	0.258458
2610507B1	6.743516539	2.774409	0.411419
Gm20830	6.737969807	3.172205	0.470795
Cbx5	6.72323333	2.353671	0.35008
Rbm5	6.716200379	2.005577	0.298618
Syne1	6.714385836	3.085098	0.459476
Pgm2	6.70579735	2.272197	0.338841
Cpeb4	6.696891244	2.685942	0.401073
Ccnl2	6.688524846	3.22348	0.481942
Mmp14	6.66398646	2.082831	0.31255
Ubr5	6.662829537	1.091543	0.163826
Cnot3	6.639389703	2.78784	0.419894
Fdft1	6.627632479	2.796627	0.421965
Pgk1	6.623159554	2.564276	0.387168
Zscan26	6.613483841	3.102548	0.469125
Omg	6.611203213	1.301685	0.196891
Kmt2c	6.598562406	1.10437	0.167365
Gm28035	6.591210986	2.981601	0.45236
Dnajc18	6.591024553	2.114081	0.320752
Atp6ap1	6.572507012	2.500984	0.380522
Rab31	6.567251066	2.497278	0.380262
Acss1	6.554844668	1.990908	0.303731
Ppp1r12a	6.553409175	2.251678	0.343589
Pdia3	6.552550207	1.929581	0.294478
Shisa9	6.544700998	3.24382	0.495641
Cdkn1b	6.526060326	2.296356	0.351875
Pbx1	6.509239665	2.62625	0.403465
Cd63	6.436302621	2.895168	0.449819
Rpl10a	6.42157264	2.552578	0.3975
Idh3g	6.420082964	2.926265	0.455799
Ppargc1a	6.416077694	1.241308	0.193468
Tmem144	6.398472596	2.300827	0.35959
App	6.365518776	3.02169	0.474697
Sfpq	6.354042318	1.96719	0.309597
9330159F1	6.344284299	2.345386	0.369685
Dmd	6.335951525	0.731035	0.115379
Sar1b	6.328112903	1.886714	0.298148
Klf9	6.326933224	1.265425	0.200006
Aldh1l1	6.30813424	2.546823	0.403736
Tmem184c	6.307776993	2.241393	0.355338
Ank2	6.300484579	0.883103	0.140164
Tmc7	6.291327698	1.194621	0.189884
Slc3a2	6.285532293	1.390203	0.221175
Eif2s3y	6.267999891	1.50301	0.239791

Cdh20	6.232892336	1.836371	0.294626
9330151L1	6.223037701	2.543578	0.408736
Rnf5	6.209924795	2.755864	0.443784
Lpcat3	6.203478961	2.63042	0.424023
Znrf3	6.19827167	1.730025	0.279114
Atl3	6.180643989	1.602744	0.259317
Nsmf	6.169416405	2.593677	0.420409
Erdr1	6.153263315	1.930653	0.313761
Akap11	6.131926397	1.231037	0.200759
Srrm1	6.121898857	2.857215	0.46672
Xpr1	6.0982619	0.505714	0.082927
Rbbp6	6.089924609	2.006082	0.32941
Hnrnpa3	6.086644785	0.995537	0.163561
Tspyl2	6.060138369	2.51608	0.415185
Rnf24	6.038007409	1.881583	0.311623
Dstn	6.02691143	2.460527	0.408257
Dpp8	6.026522678	1.985338	0.329433
Soat1	6.024589759	1.227161	0.203692
Amy1	6.020640739	1.577903	0.262082
Arl8b	6.01223871	2.875144	0.478215
Neo1	6.001599596	1.716323	0.285978
Hes5	5.999150193	2.994632	0.499176
Eef2	5.997072682	2.811297	0.468778
Cdc37l1	5.985822061	1.792001	0.299374
Mdm4	5.984528854	0.410392	0.068576
Rpl4	5.967513655	2.785925	0.466848
Msi2	5.952841588	1.703303	0.286133
Ktn1	5.951294686	2.377287	0.399457
Zeb1	5.93370675	1.188847	0.200355
Pkm	5.928977554	2.587512	0.436418
Lfng	5.924874782	2.253369	0.380323
Hipk2	5.918776283	1.3005	0.219725
Clk3	5.913121743	2.904805	0.491247
Pcdh7	5.895551398	1.588387	0.269421
Pik3r1	5.891914118	1.480359	0.251253
Luc7l2	5.838479875	2.00122	0.342764
Adhfe1	5.834732711	1.43891	0.246611
Capn7	5.821381196	2.11572	0.36344
Gm12359	5.807320392	2.165598	0.372908
Sall2	5.796129142	0.987087	0.170301
Ptpra	5.772728467	1.228735	0.212852
Med13	5.754541337	1.393021	0.242073
Lxn	5.715272523	2.358252	0.412623
1500009C(	5.710227766	2.432148	0.425928
Chkb	5.707750803	2.47789	0.434127
Slmo2	5.704164628	1.958545	0.343354
Ralgds	5.703028378	2.069744	0.36292

Bhlhe40	5.688024263	2.576456	0.452962
Xpo1	5.67282288	1.643993	0.289802
Srsf11	5.670009765	1.92754	0.339954
Nedd4	5.660570348	1.931838	0.34128
Senp6	5.643489236	1.789104	0.317021
2810403A(	5.632062473	0.68156	0.121014
Abat	5.61949247	2.459384	0.437652
Mga	5.614928884	1.203079	0.214264
Npas3	5.606467417	0.741422	0.132244
Egfr	5.591492273	1.801515	0.322189
Slc25a3	5.586966751	1.519865	0.272038
Set	5.58408463	1.160544	0.207831
Rab10	5.582384208	0.827963	0.148317
Prpf4b	5.56707395	1.73703	0.312018
Uqcrfs1	5.566065371	2.481569	0.445839
Nars	5.557704249	2.7491	0.494647
Sirpa	5.545739186	1.955497	0.352612
Pea15a	5.542286525	1.850169	0.333828
Kank1	5.53950167	2.299571	0.415122
Tor1aip1	5.530880286	2.664239	0.481703
Gxylt1	5.517828452	2.234015	0.404872
Vcam1	5.511977786	2.342054	0.424903
Zbtb20	5.505138905	2.281624	0.414454
Pnp	5.495891564	2.455334	0.446758
Ttc14	5.494387562	0.864439	0.157331
Cox5a	5.480197547	1.85264	0.338061
Fnbp1	5.468397793	0.439773	0.080421
Cntfr	5.459517133	2.546127	0.466365
Ubxn4	5.448727751	2.54753	0.467546
Fat3	5.431705998	0.776436	0.142945
Phyhd1	5.423830604	2.339023	0.431249
Zfc3h1	5.400469567	1.995131	0.369437
Csde1	5.39392019	2.610376	0.483948
Cry2	5.3890497	2.349306	0.435941
Rbm12b2	5.386293274	1.539905	0.285893
Eif5	5.382051608	1.999583	0.371528
Gm17555	5.376298848	2.315943	0.430769
Scarb2	5.364969999	1.596033	0.297491
Atp13a5	5.361531867	1.831955	0.341685
Pafah1b1	5.355554683	2.155751	0.402526
Tubb2b	5.336006134	2.178001	0.408171
Sqle	5.325571777	2.463855	0.462646
Gm28986	5.320272666	2.298134	0.431958
Idh1	5.310031794	1.923015	0.362148
Aldoa	5.302934514	1.972827	0.372026
Hook3	5.295316854	1.292776	0.244136
Mfn1	5.291132245	1.522013	0.287653

Cldn10	5.287400282	2.023342	0.382672
Prpf19	5.27859492	1.683352	0.318902
Tmbim6	5.277508182	2.448522	0.463954
Eif3a	5.270336923	1.398451	0.265344
Hif1a	5.267970744	1.26376	0.239895
Capn2	5.236167901	1.943515	0.371171
Dock4	5.225886703	0.612115	0.117131
Arap2	5.224947922	1.493416	0.285824
Mfap1a	5.207576889	2.3428	0.449883
Usp24	5.206335944	1.346815	0.258688
Sf3b1	5.205025776	0.41369	0.079479
Ppm1g	5.198771403	2.020935	0.388733
Prdx1	5.180226519	1.529565	0.29527
Srgap3	5.175927883	1.840501	0.355589
Igsf1	5.170956351	1.450751	0.280558
Acbd5	5.168700336	1.055612	0.204232
Oxct1	5.163103623	1.291657	0.250171
Utrn	5.157507059	0.199976	0.038774
Eno1	5.139541094	1.618839	0.314977
Dnm1l	5.130588228	1.890768	0.368529
Lnpep	5.121999137	1.384698	0.270343
Capns1	5.11554328	1.729782	0.338142
Sdc2	5.114577233	1.21813	0.238168
Fam193b	5.110688773	1.926004	0.376858
Tsc2	5.102326779	1.942529	0.380714
Gtf3c2	5.096389168	2.403204	0.47155
Dtx3	5.076351514	2.473203	0.487201
Prpf39	5.061695397	1.9406	0.383389
Fbxo44	5.044296819	2.242454	0.444552
Cep85l	5.042321252	1.238395	0.2456
Inppl1	5.041757666	2.145888	0.425623
Tab2	5.025761825	1.483188	0.295117
Gm21750	5.01302113	1.720356	0.343177
Elovl2	5.011620481	1.201635	0.23977
Col9a3	5.011515444	2.361647	0.471244
Ctnna1	5.009221229	2.172417	0.433684
Gm2163	4.998246859	2.157449	0.431641
Usp11	4.997632947	2.344358	0.469094
Smc3	4.991174864	1.815025	0.363647
Rbm39	4.990097098	1.462225	0.293025
Rsrc2	4.990069139	0.687342	0.137742
Aldh6a1	4.97703431	1.766605	0.354951
Gm12940	4.975091928	2.042304	0.410506
Itgb8	4.973741949	0.852527	0.171406
Atf1	4.973211077	1.640812	0.32993
Ssbp1	4.962834232	1.643291	0.331119
Hsd17b11	4.95538598	1.153093	0.232695

2210016F1	4.95492346	1.942701	0.392075
Ndufc2	4.947554216	1.844646	0.37284
Nrcam	4.938489818	0.668364	0.135338
Slc25a18	4.938257688	1.841075	0.372819
Fmo1	4.931600861	1.573735	0.319112
Nipbl	4.918399276	1.285557	0.261377
Prex2	4.915306859	1.118408	0.227536
Fbxl3	4.906170416	0.601522	0.122605
Wsb1	4.903328218	1.326102	0.270449
Socs7	4.893992001	1.519748	0.310533
Nfat5	4.887517009	1.280381	0.26197
Tlcd1	4.883357434	0.785689	0.160891
Olig2	4.882625065	1.769167	0.362339
Ssfa2	4.880513297	1.972192	0.404095
Gna12	4.868685757	1.878744	0.385883
Tuba1b	4.853514826	2.023909	0.416999
Sesn3	4.844458914	1.477076	0.3049
Ak3	4.841609182	1.717687	0.354776
Elmo2	4.83843478	2.087844	0.431512
Tmem106l	4.836556931	0.368998	0.076294
Fmnl2	4.829101502	1.92209	0.398022
Desi2	4.821631775	0.863117	0.179009
Mertk	4.818944236	1.86548	0.387114
Chfr	4.799296438	2.011785	0.419183
Rev3l	4.794527609	0.448749	0.093596
Zmiz1	4.790933331	2.297546	0.479561
Atp11b	4.783767794	1.192979	0.249381
Ralgapa1	4.779791852	1.404537	0.293849
Caml	4.7783522	2.376737	0.497397
Tlr3	4.776876993	1.680182	0.351732
Birc2	4.772315942	2.193166	0.45956
Ankrd28	4.770992239	1.483414	0.310924
Htra1	4.757423501	1.739099	0.365555
Fgd6	4.755226095	1.373763	0.288895
Slc29a3	4.748309239	0.534757	0.112621
Pcdh10	4.724745869	1.761926	0.372915
Sesn1	4.724027409	2.293271	0.485448
Atp5g3	4.72317076	1.435194	0.303862
Ctso	4.719627864	2.199076	0.465943
Pdlim5	4.710980397	1.537665	0.3264
Sorbs1	4.710470186	1.689818	0.358736
Nktr	4.702657766	1.262936	0.268558
Abca1	4.702393687	1.543711	0.328282
Paqr7	4.699772081	1.321713	0.281229
Rtn4	4.696642347	1.511048	0.321729
Celsr2	4.692686767	1.535775	0.32727
Slc22a17	4.6854506	1.013465	0.2163

Klhl24	4.679412987	2.250298	0.480893
Rab6a	4.672803262	1.402998	0.300248
Pot1b	4.64934266	0.548779	0.118034
Gm28217	4.635926166	2.247665	0.484836
Gm884	4.604436972	1.977021	0.429373
Dynlt3	4.578299416	1.661689	0.362949
Rbm25	4.566779133	1.765719	0.386644
Ugp2	4.56563078	2.0166	0.441691
Srebf1	4.558494844	1.477821	0.324191
Gm20831	4.554291774	2.081522	0.457046
Gm10561	4.55379233	2.260225	0.496339
Ctdsp2	4.552954131	0.991581	0.217788
Rb1	4.552654112	1.897395	0.416767
Drp2	4.546553842	1.963583	0.431884
Gli1	4.53977789	1.171071	0.257958
Ifi27	4.537251846	1.753117	0.386383
Atg3	4.531617981	1.92374	0.424515
Ppp6r3	4.530292069	2.037934	0.449846
Gtf2i	4.523990516	1.732053	0.382859
Degs1	4.505019676	1.323343	0.293748
Tcf25	4.502157441	2.027434	0.450325
Immt	4.495105855	1.735458	0.386077
Huwe1	4.489779891	0.918438	0.204562
Gak	4.484031691	2.004286	0.446983
Cntn1	4.483950822	1.356163	0.302448
Araf	4.478399471	1.253193	0.279831
Rbm6	4.476482256	2.049534	0.457845
Usp8	4.474887441	1.667332	0.372597
Vps13b	4.47271796	1.437401	0.321371
Tax1bp1	4.463187197	1.644682	0.368499
Lrrc16a	4.46262047	1.924535	0.431257
Ccnl1	4.461352702	1.958536	0.439
Mapk4	4.450805524	1.559971	0.350492
Nras	4.44717838	2.081529	0.468056
Mbnl2	4.432454694	0.737757	0.166444
Ece1	4.422818646	1.729354	0.391007
Kmt2e	4.41503681	1.311858	0.297134
Tpt1	4.413349834	1.828237	0.414252
Slitrk2	4.411369456	0.86952	0.197109
Wnk1	4.410290072	0.850168	0.192769
Plxnb1	4.409092523	1.957051	0.443867
Nfia	4.40767772	1.212535	0.275096
Phlpp1	4.396755367	1.349986	0.307041
D830044I1	4.392764906	2.040691	0.464557
Cdc42	4.389240735	1.310843	0.298649
Lrpap1	4.387050215	1.137892	0.259375
Hiat1	4.377332221	1.962908	0.448426

Hnrnpdl	4.376096636	1.572635	0.359369
Idh3b	4.37291058	1.635523	0.374012
Plcb4	4.367518681	1.761907	0.403411
Eif4g2	4.362022428	1.650533	0.378387
Ccar1	4.352770659	1.754166	0.403
Rorb	4.341435679	1.293884	0.298031
Adi1	4.337290944	1.93048	0.445089
Alcam	4.336726569	0.844043	0.194627
Gm8300	4.333685719	1.324577	0.305647
Nbea	4.332054218	2.105668	0.486067
6820431F2	4.329841551	0.811113	0.187331
Dzip1	4.329615378	1.335713	0.308506
Nhsl1	4.329558956	1.823678	0.421216
Cd38	4.329199799	0.97405	0.224995
Rpl7a	4.327788058	1.334927	0.308455
Ubn2	4.326831219	1.177285	0.272089
Chd4	4.322954436	2.119803	0.49036
Lgmn	4.322082577	1.206552	0.27916
Rnf20	4.317830703	0.785748	0.181977
Sox6	4.312765889	2.02968	0.470621
Ptpdc1	4.308401538	2.001789	0.464625
Spred1	4.307978808	1.382447	0.320904
Emc3	4.307568268	1.185603	0.275237
Abcc5	4.292262021	1.09669	0.255504
Ywhab	4.27568844	1.831371	0.428322
Ptp4a2	4.272041356	1.304894	0.30545
Anapc5	4.260613278	1.375667	0.32288
Zc3h15	4.259874915	1.255173	0.29465
Sf3a1	4.25148545	1.927867	0.453457
Muc4	4.23923911	0.644492	0.15203
Kpnb1	4.238492888	1.239637	0.292471
Gatm	4.236422325	1.538434	0.363145
Tmx1	4.230906248	1.122423	0.265291
Pdcd5	4.229361474	1.575231	0.372451
Vcl	4.216777109	1.501264	0.356022
Trip11	4.216310262	1.199331	0.28445
Cc2d2a	4.210472403	1.452604	0.344998
Cnot1	4.201346487	1.230458	0.292872
Gna13	4.19732901	1.317732	0.313945
BC005537	4.185332028	1.600185	0.382332
Abcd3	4.167969559	1.066174	0.255802
Nf1	4.162504091	1.319643	0.317031
Atraid	4.157727955	1.991412	0.478967
Crebbp	4.156021095	1.27851	0.307628
Esco1	4.155761257	0.671581	0.161602
St5	4.155072505	1.784492	0.429473
Kat6a	4.154011608	1.908718	0.459488

Gli2	4.152821552	1.471	0.354217
Tcf20	4.141790351	1.715753	0.414254
Rpl10	4.139798689	1.071239	0.258766
Zfp3	4.130602385	1.839398	0.44531
Reps1	4.12399977	1.537567	0.372834
Kcnc4	4.120069126	1.938931	0.470606
Rpsa	4.112910763	1.721152	0.418475
Ggnbp2	4.110367788	1.365257	0.332149
Ak4	4.102088441	1.523312	0.37135
Insr	4.101795201	0.989589	0.241258
Cxcl14	4.09700428	1.623854	0.396352
Rcn2	4.096209098	0.920528	0.224727
Hivep1	4.09554673	1.822763	0.44506
Iqsec1	4.095226108	1.249723	0.305166
Zmym2	4.086814644	1.703215	0.416759
Smpd2	4.085790801	1.350132	0.330446
Ptges3	4.083301698	1.557146	0.381345
Zfp281	4.077117655	2.008749	0.492689
Helz	4.065040117	1.377416	0.338844
Ankrd40	4.054398782	1.166495	0.287711
Gprc5b	4.052860103	1.47431	0.36377
Cirh1a	4.043896594	1.953321	0.483029
Rfx3	4.031057637	1.050152	0.260515
Itpr2	4.028846193	0.912715	0.226545
Sema4a	4.028200973	1.394855	0.346272
Hspa4l	4.026255436	1.687606	0.41915
Phyhipl	4.026047202	0.833708	0.207079
Ankrd52	4.025956108	1.610231	0.399962
Epas1	4.01640447	1.316945	0.327892
Gm20521	4.012444031	1.724374	0.429757
Asap1	4.010888657	1.413297	0.352365
Dusp11	4.009481617	0.601967	0.150136
Ep300	4.008597942	0.892318	0.222601
Klhl5	4.005134246	1.307656	0.326495
Robo1	4.004479918	1.615425	0.403404
Setd5	3.998820369	1.650958	0.412861
Bod1l	3.990065989	1.27868	0.320466
Tbc1d5	3.985431931	1.219687	0.306036
Gaa	3.980113901	1.661544	0.417462
Sned1	3.960615419	1.917984	0.484264
Gpatch8	3.958933127	1.119871	0.282872
Fasn	3.958406572	1.968871	0.49739
Eif4g3	3.947309398	0.431259	0.109254
Prkar1a	3.942648482	1.781481	0.451849
Slc7a11	3.9369106	1.185191	0.301046
Mfsd2a	3.930009344	0.791049	0.201284
Ptprs	3.92809475	1.26642	0.322401

Aifm3	3.921319563	1.028408	0.262261
Gm26798	3.920903074	1.855703	0.473285
Rere	3.918755324	1.562754	0.398788
Fus	3.917970919	1.380419	0.35233
Bglap3	3.917749473	1.242391	0.317119
Ino80d	3.911506341	1.276709	0.326398
Phf21a	3.910370019	0.836057	0.213805
Calm2	3.908610293	1.939823	0.496295
Dlst	3.903860511	1.895561	0.485561
Itpkb	3.901281358	1.192628	0.305702
Gm5108	3.899122098	1.687966	0.432909
Zfp280c	3.894036716	1.902447	0.488554
Daam1	3.876132822	1.879711	0.484945
Hsp90aa1	3.864441337	0.931635	0.241079
Ube2d3	3.862896507	1.218553	0.31545
Scamp5	3.861486299	1.599623	0.41425
Trac	3.855238851	1.751397	0.45429
Limk2	3.847997603	1.183791	0.307638
Pan3	3.84672408	1.833424	0.47662
Reep5	3.843184406	0.877691	0.228376
Per3	3.843042915	1.188195	0.309181
Pcmtd1	3.842279155	0.898206	0.233769
Ctage5	3.837957307	1.433647	0.373544
Gprasp1	3.837764077	1.258635	0.32796
Chst10	3.835304113	1.255977	0.327478
Nsd1	3.829495275	0.310626	0.081114
Ash1l	3.823875394	1.420933	0.371595
Ube2l3	3.815989116	1.787828	0.46851
Atp2b2	3.801717001	1.30854	0.344197
Senp1	3.798335715	1.02317	0.269373
Nr1d2	3.796491146	1.731625	0.456112
Chpt1	3.796237678	0.673149	0.17732
Kcnk1	3.794097083	0.880257	0.232007
Ccdc141	3.793151496	1.049065	0.276568
Dst_1	3.791295778	1.630824	0.430149
Herpud1	3.784955152	1.885833	0.498244
Iqck	3.77925669	0.762216	0.201684
Cfl1	3.777452153	1.143847	0.302809
4632427E1	3.776359594	1.729635	0.458017
Marf1	3.767901287	0.367043	0.097413
Cd9	3.765887893	1.41119	0.37473
Dpy19l4	3.765668392	0.912762	0.24239
Csnk1a1	3.764656454	1.530288	0.406488
Akap9	3.762777216	0.566817	0.150638
Vps54	3.760254893	1.709213	0.454547
Lrrc58	3.753719394	0.646101	0.172123
Acap2	3.744432312	1.538212	0.4108

Cnep1r1	3.738785001	1.689981	0.452013
Ak2	3.734530855	1.531097	0.409984
Fubp1	3.73126454	1.035865	0.277618
Mfn2	3.730699212	1.269146	0.34019
Selt	3.728041292	1.542824	0.413843
Pcf11	3.726506556	1.261123	0.33842
Gpt2	3.725748262	0.993689	0.266709
Npat	3.72381616	1.643791	0.441427
Fut9	3.703060112	1.178562	0.318267
Lifr	3.701153723	1.486974	0.40176
Map2k7	3.696235499	1.585733	0.429013
Dgkb	3.68961713	1.499035	0.406285
Xrn2	3.689339532	1.416603	0.383972
Wwtr1	3.675985009	1.679222	0.456809
Csdc2	3.674137811	1.333303	0.362889
Fam214b	3.669038621	1.444941	0.39382
Lrrc4c	3.658414404	1.567175	0.428375
Ids	3.657182386	1.424433	0.389489
Acot11	3.654489588	1.646235	0.450469
Rpl17	3.653360576	1.442163	0.39475
Bmpr1b	3.652536107	1.059212	0.289994
Frs2	3.637053373	1.81838	0.49996
Fam168b	3.633776324	1.738018	0.478295
Aco2	3.629921597	1.091905	0.300807
Prodh	3.623415067	1.488543	0.410812
Cyfip1	3.610597369	1.381861	0.382724
Kcna2	3.602326147	0.960383	0.266601
Plce1	3.600560915	1.376514	0.382305
Gm11033	3.600556628	1.65631	0.460015
Tcp11l1	3.597699385	1.271277	0.353358
Atf2	3.596917372	1.476622	0.410524
Wasl	3.589426201	0.991175	0.276138
Actr2	3.587612193	1.178022	0.328358
Adgrl3	3.58667096	1.507127	0.420202
Zfp318	3.586362186	1.562996	0.435817
Tia1	3.58393614	0.72929	0.203489
Mtx3	3.582382204	1.097273	0.306297
Epb4.1l2	3.577784254	1.494389	0.417686
Hnrnpl	3.576236868	1.064753	0.29773
Puf60	3.57109127	1.780308	0.498533
Srpk2	3.569122094	1.534696	0.429992
Arrb1	3.561687653	0.676864	0.19004
Gm26804	3.560171842	1.202072	0.337645
Tmod2	3.560036882	0.784868	0.220466
Arl6ip1	3.559816838	1.690461	0.474873
Thrsp	3.553421813	1.721458	0.484451
Bbs2	3.551585569	1.585213	0.446339

Vps4a	3.55142477	1.425589	0.401413
Mrpl16	3.551404935	1.620355	0.456257
Notch3	3.541838702	1.761981	0.497476
Btbd3	3.541836579	0.975014	0.275285
Map1a	3.539003237	1.203223	0.339989
Antxr1	3.536669312	0.50786	0.143598
Irgq	3.532397481	1.279488	0.362215
Ddx3x	3.529256668	0.517038	0.1465
Mir99ahg	3.529167385	1.719147	0.487126
Vdac1	3.517019136	1.153282	0.327915
Atxn2l	3.511858156	0.888721	0.253063
Wrn	3.511264963	1.476829	0.420598
AW82207З	3.51057051	1.473209	0.41965
Rhoq	3.510099855	1.750708	0.498763
Fnbp1l	3.509184029	0.801674	0.22845
Stk24	3.501566657	1.646452	0.470204
Tmem170l	3.501323941	0.920513	0.262904
Hnmt	3.495027458	1.686431	0.482523
Sqstm1	3.493189426	1.150323	0.329305
Brd4	3.491267934	1.151984	0.329962
Ptprm	3.488903543	0.802885	0.230125
Pogz	3.487914328	1.272156	0.364733
Mgst1	3.484091529	1.73095	0.496815
Hnrnpu	3.483108239	1.34029	0.384797
Mrpl21	3.479048505	1.664154	0.478336
Ncstn	3.478723403	1.604722	0.461296
Trim44	3.476230654	1.217302	0.350179
Lrrc59	3.469834993	1.027676	0.296174
Pcdh17	3.466601317	1.649073	0.475703
Rimklb	3.464796993	0.909339	0.262451
Gm9801	3.456471151	1.482737	0.428974
Bmpr2	3.455673552	0.692228	0.200316
Fuca1	3.452759722	1.1493	0.332864
Atp6v0a1	3.441194432	1.570116	0.456271
Kmt2d	3.437151461	0.463226	0.13477
Srgap1	3.434288671	0.438579	0.127706
Dalrd3	3.431680555	0.98837	0.288013
Hmgb1	3.431081151	1.320654	0.384909
Supt6	3.426761869	0.562847	0.16425
Erlin2	3.425223854	0.918187	0.268066
Dtx4	3.41228961	1.459487	0.427715
Arid4a	3.40971569	0.782724	0.229557
Tsr1	3.401656021	1.038142	0.305187
Kat2a	3.400466959	1.19405	0.351143
Cbx3	3.399541245	0.770797	0.226736
Hsdl2	3.396042325	1.109214	0.32662
Prdm2	3.396028204	0.35949	0.105856

Fam222b	3.395876415	1.166366	0.343465
Slc35b1	3.395224926	1.370645	0.403698
Sos1	3.38834333	0.992218	0.292833
Arsb	3.386873391	1.117903	0.330069
Papola	3.383011401	0.997196	0.294766
Muc6	3.382990188	1.561871	0.461683
Ckap5	3.372623132	1.344939	0.398781
Pccb	3.371934221	1.680663	0.498427
Prrt1	3.364906659	1.153035	0.342665
Cdc40	3.364639315	1.286307	0.382301
Nckap5	3.364338337	1.253426	0.372562
Itsn2	3.361058694	0.905905	0.26953
Nisch	3.356381003	0.839725	0.250188
Gdi2	3.355320201	1.355467	0.403975
Tapt1	3.354493166	1.029792	0.306989
Ctsb	3.346005528	0.219976	0.065743
Fbxo9	3.344097851	0.887288	0.265329
Rpl18a	3.343813944	1.223389	0.365866
Mycbp2	3.338128371	0.601447	0.180175
Usp54	3.33149043	1.298331	0.389715
Ppfia1	3.323981406	1.374223	0.413427
Tmem164	3.321949224	1.632494	0.491427
4933407K1	3.320370419	1.415156	0.426204
Fstl1	3.320360271	1.428681	0.430279
Chuk	3.31877071	1.095677	0.330145
Cnot4	3.311880327	0.605179	0.18273
Pias1	3.310195612	0.883681	0.266957
Hnrnph3	3.309631802	1.434619	0.433468
Lsm6	3.308493186	1.151029	0.347901
Slc15a2	3.306157301	0.78228	0.236613
Rps23	3.295693477	1.375477	0.417356
Pura	3.292663881	1.373008	0.41699
Cdh2	3.291773058	1.260049	0.382787
Hif3a	3.290493327	1.54954	0.470914
Grm3	3.288141352	1.337137	0.406654
Smap1	3.281322108	1.087078	0.331293
Baz2b	3.273975537	1.02873	0.314214
Itfg1	3.273325771	0.111611	0.034097
Atg9a	3.264207845	1.479014	0.4531
Snrnp200	3.263765317	1.129168	0.345971
Clptm1	3.26250076	1.238223	0.379532
Gabpa	3.243717647	1.300845	0.401035
Rad21	3.239233088	0.681193	0.210295
Rab11fip2	3.23661153	1.388367	0.428957
Eif4a1	3.234777286	0.851845	0.26334
Mapre2	3.230109402	1.021762	0.316324
Rplp2	3.227726591	1.310646	0.406059

Gm21833	3.226644684	1.384866	0.429197
Otud5	3.224290195	1.416643	0.439366
Bfar	3.221829947	1.45488	0.45157
Kmt2a	3.216264101	0.520035	0.161689
Adcy8	3.214036462	1.125056	0.350045
Hnrnpd	3.209683904	1.576014	0.491018
Gm4477	3.208844829	1.544379	0.481288
Zfp521	3.207589031	1.163704	0.362797
AA465934	3.204195295	1.229972	0.383863
Fnbp4	3.199664762	1.34425	0.420122
Taf1d	3.198101612	0.946084	0.295827
Chd1	3.197803399	1.18771	0.371414
Zfp384	3.195963323	1.079401	0.337739
Rpn2	3.188163345	0.901651	0.282812
Fgfr1	3.182754166	1.208092	0.379574
Tug1	3.180701549	1.203415	0.378349
Dcun1d5	3.179878877	1.268823	0.399016
Bms1	3.169413075	1.040575	0.328318
Otx2	3.158634746	1.492493	0.472512
Nfatc3	3.158251843	1.079647	0.34185
1700029F1	3.155897789	0.687876	0.217965
Itga6	3.155473361	0.988798	0.31336
Lair1	3.154425281	1.289281	0.408721
Acp2	3.153932177	0.9979	0.316399
Srsf2	3.149824883	1.391472	0.441762
Zgpat	3.146366843	1.194687	0.379704
Sgip1	3.145527574	0.748807	0.238055
Tspan7	3.144724568	1.001388	0.318434
Ptpn4	3.144484169	1.103098	0.350804
Taf1	3.14185604	1.1035	0.351226
Zfp36l1	3.141708053	1.343919	0.427767
Dtx1	3.141656025	0.87271	0.277787
Usp32	3.140979637	1.324801	0.421779
Bhlhe41	3.138933257	1.558135	0.49639
Ttc1	3.133491315	0.71111	0.226938
Fxr1	3.13190319	0.98062	0.313107
Gabrg1	3.130699118	1.227411	0.392057
Kat6b	3.130434511	0.520321	0.166214
Elk1	3.125004409	0.898716	0.287589
Hsph1	3.124025098	1.039238	0.33266
Zfp687	3.124013514	1.054524	0.337554
Gm14565	3.114044296	0.870225	0.279452
Ybx1	3.105983302	1.157817	0.37277
Grk4	3.099755526	1.097213	0.353968
Spcs3	3.097380487	1.026431	0.331387
Il33	3.082223016	0.825675	0.267883
Tef	3.069951555	1.113531	0.362719

Lrp1b	3.059737515	0.604544	0.19758
Dnaja2	3.057993643	1.33049	0.435086
Zfp329	3.054608424	1.356949	0.44423
Sp110	3.054421458	0.798281	0.261353
Lonp2	3.043205304	1.458302	0.479199
Rpl11	3.036986923	1.07187	0.352939
Hmgcr	3.033248901	0.876467	0.288953
Zfp608	3.033236282	1.420505	0.468313
Stim1	3.032196835	1.187891	0.391759
Caskin1	3.029704869	0.45114	0.148906
Dock7	3.026605929	0.927422	0.306423
Alg5	3.026006157	1.402832	0.463592
Paqr4	3.022525607	1.334802	0.441618
Chd6	3.021427934	0.720801	0.238563
Vamp2	3.021326709	1.484423	0.491315
Oat	3.021280638	1.008841	0.333912
Ivns1abp	3.018091971	1.504419	0.498467
Psmd11	3.017082773	1.335495	0.442645
Fam92a	3.014236798	0.947557	0.314361
Ylpm1	3.014030404	0.951381	0.315651
Ankhd1	3.009864327	0.30958	0.102855
Efr3b	3.009002547	0.929227	0.308816
Nln	3.008963936	1.137968	0.378193
Mettl23	3.007391801	1.26005	0.418984
Flrt2	2.994188678	1.340121	0.447574
Slc30a10	2.990144244	1.48457	0.496488
Acsf2	2.983019195	1.474218	0.494203
Tm9sf2	2.981467948	1.097241	0.36802
Gns	2.980669305	1.152492	0.386655
Pip4k2a	2.975021741	0.826507	0.277816
Mboat2	2.973572882	1.107968	0.372605
Tmem131	2.972841003	1.402737	0.471851
Atxn1	2.972751685	0.973887	0.327604
Gm6483	2.97213233	1.261483	0.424437
Tmem176b	2.969704137	1.473291	0.496107
Hspa9	2.966981492	1.156741	0.389871
Xpnpep1	2.966730166	1.280822	0.431729
Dclk1	2.960163299	1.251838	0.422895
Bmp2k	2.959335067	1.467991	0.496054
Adgrb3	2.956935701	1.433878	0.48492
Gm14717	2.956421404	1.313175	0.444177
Hp1bp3	2.953811669	1.136618	0.384797
Rnf215	2.953282518	1.207648	0.408917
Lats1	2.953007426	0.876468	0.296805
Bptf	2.94980257	0.679593	0.230386
Zranb2	2.944715921	0.820233	0.278544
Gsk3b	2.94300299	0.849814	0.288757

Fah	2.942430064	0.969231	0.329398
Ubr2	2.940213776	0.958909	0.326136
Pabpc1	2.935977637	0.531683	0.181092
Me1	2.935352684	1.257009	0.428231
Magi2	2.929037775	1.338941	0.457127
Nlgn3	2.921948182	0.228902	0.078339
Cpeb2	2.919900413	0.558311	0.191209
Slc30a1	2.915478064	1.423271	0.488178
Sbf2	2.909766076	1.099789	0.377965
Lpp	2.909303444	1.047275	0.359975
Sec61a1	2.906441609	0.792605	0.272706
Nnt	2.905925785	1.101742	0.379136
Chka	2.902296713	1.304528	0.449481
Sptan1	2.901615246	0.443702	0.152915
Fabp5	2.898456451	1.361859	0.469857
Sin3a	2.896709065	0.962078	0.332128
Enpp5	2.894870043	1.024537	0.353915
4933431E2	2.890301255	1.111918	0.384707
Cops2	2.887546017	0.715993	0.247959
Cipc	2.882824385	1.393135	0.483254
Bcl2	2.881543138	0.744296	0.258298
Etfa	2.878796527	0.95712	0.332472
Pdha1	2.878703036	1.030596	0.358007
Phf12	2.878169434	1.27217	0.442007
Klhl17	2.876886527	1.231858	0.428191
Rpl23a	2.875522995	1.132879	0.393973
Zmat3	2.872078553	1.100462	0.383159
Id2	2.871856857	1.132171	0.39423
Gm10172	2.87030948	1.243544	0.433244
Adgra1	2.86991914	1.228116	0.427927
Atrx	2.869290675	0.775945	0.270431
Gramd3	2.867752746	1.29491	0.451542
Nfasc	2.867619963	1.074474	0.374692
Actn4	2.865383627	1.055405	0.368329
Fbxo18	2.8631874	1.0862	0.379367
Plxdc2	2.863167837	1.331571	0.465069
Itpr1	2.861909276	0.937292	0.327506
Ccdc47	2.860087759	0.469932	0.164307
Psma4	2.855696033	1.209558	0.42356
Dennd5a	2.853740802	0.67128	0.235228
Sltm	2.851704524	1.091134	0.382625
Tcn2	2.850448137	1.058008	0.371173
Ghr	2.847386567	0.947332	0.332702
Plekhb1	2.844792019	0.755971	0.265739
Gm4491	2.839077544	1.410296	0.496745
Itgb1	2.838859774	1.403647	0.494441
Tardbp	2.83853356	0.924806	0.325804

Pfn1	2.834509613	1.30609	0.460782
Ppt1	2.834153718	1.413503	0.498739
Msi1	2.830011111	1.203391	0.425225
Psg16	2.824977882	0.823571	0.291532
Sp3	2.820983623	0.717739	0.254429
Zfp395	2.819356671	0.821188	0.291268
Nrarp	2.814096847	0.869937	0.309135
Tcea1	2.810495496	1.092307	0.388653
Gtpbp4	2.802938878	1.38926	0.495644
Slc9a3r1	2.802131792	1.25268	0.447045
Cd200	2.797791947	1.183771	0.423109
Gm21874	2.791621282	0.892593	0.31974
Rora	2.789860808	0.877117	0.314394
Sptbn1	2.785766666	0.972985	0.34927
Med12	2.782308651	0.840514	0.302092
Adamts9	2.781942759	1.034335	0.371803
Ogdh	2.778170983	1.353703	0.487264
Impact	2.776608519	1.280029	0.461005
Cdk13	2.773766728	1.357296	0.489333
Tbc1d32	2.773359392	1.164905	0.420034
Prkar2a	2.772914651	1.125455	0.405874
Tnks2	2.772299983	1.240153	0.447337
Zfp263	2.771078621	1.181257	0.426281
Chn1	2.769096161	0.633273	0.228693
Mrpl37	2.766548955	1.307469	0.472599
Snapc3	2.761443366	1.364293	0.494051
Prrc2a	2.755392015	0.903275	0.327821
Gm20917	2.752042022	1.265753	0.459932
RP23-97A1	2.748336329	1.329822	0.483864
Hmgcs1	2.748027259	0.992414	0.361137
Marcks	2.74757195	0.63858	0.232416
Snx32	2.743712779	0.946472	0.34496
E330020D1	2.742130171	1.23065	0.448793
Vcan	2.740219651	1.158515	0.422782
Gm20721	2.73820627	0.834959	0.304929
Slc22a4	2.732870088	1.208436	0.442186
Ranbp2	2.730479294	1.309011	0.479407
Arhgef11	2.724380317	0.901757	0.330995
Gli3	2.721947259	0.981	0.360404
Rdx	2.718531715	0.677397	0.249178
Ndufaf4	2.718469364	0.611878	0.225082
Krt7	2.718294503	1.095603	0.403048
Camk2n1	2.718219322	0.888075	0.326712
Ddx6	2.716728013	0.848667	0.312386
Syngap1	2.714110913	0.960218	0.353787
Anapc1	2.712953388	1.244845	0.458852
Aak1	2.709127508	1.032011	0.380938

Cep95	2.706704283	1.02503	0.378701
Kdm5a	2.705305219	0.768797	0.284181
Kdm3b	2.703534587	0.92483	0.342082
Ganab	2.699930104	0.989018	0.366313
Pou3f4	2.695295177	1.239475	0.459866
Mro	2.695057615	1.132836	0.420338
Stk17b	2.68587358	1.285729	0.4787
Atp6v1e1	2.685599546	1.066033	0.396944
Wdfy3	2.685522677	0.69028	0.257038
Sorbs2	2.683957721	0.773858	0.288327
Zfp326	2.680646294	0.734413	0.273968
Sorcs2	2.678242415	1.248701	0.466239
Zdhhc24	2.677811225	0.943873	0.352479
Prpf18	2.675900016	0.909365	0.339835
Abhd12	2.67584366	0.941627	0.351899
Myadm	2.671257827	0.931225	0.348609
Ildr2	2.670707825	0.88144	0.33004
Fat1	2.668079786	0.327672	0.122812
Ptma	2.666224959	1.030747	0.386594
Rfx7	2.665740627	0.853924	0.320333
Apbb2	2.665739961	0.696452	0.26126
Gm608	2.661650362	1.065957	0.400487
Arhgef28	2.658106957	0.812099	0.305518
Pibf1	2.654386293	1.220079	0.459646
Ndufv1	2.65360146	0.807515	0.304309
Glrx2	2.653513336	1.18052	0.444889
Bzw1	2.652530109	1.143364	0.431047
Rab9	2.64727263	0.932632	0.352299
Vta1	2.645265079	1.299096	0.491102
Ubr4	2.643397055	0.847574	0.320638
Plcb1	2.641400537	1.160434	0.439325
Ncam2	2.641023813	1.194147	0.452153
Sfi1	2.640922523	1.111983	0.421058
Atp6v1a	2.640289104	1.268714	0.480521
Gm29044	2.638423344	1.230493	0.466374
Nudt9	2.637521473	1.131395	0.428962
Rnpc3	2.633053859	0.512558	0.194663
Dcp2	2.632324993	0.926327	0.351904
Ehbp1	2.632039743	1.12273	0.426563
Fzd3	2.630961156	1.064603	0.404644
Mrpl18	2.629539728	1.286974	0.489429
Akap8	2.629521393	0.688112	0.261687
Zcrb1	2.628506343	1.094133	0.416257
Smg5	2.627866421	1.176121	0.447557
Atp6v0b	2.625652966	0.612232	0.233173
Stip1	2.619074308	1.032531	0.394235
Sumo1	2.613066309	1.09407	0.418692

Itgav	2.607836157	0.843649	0.323506
Apln	2.605550287	1.207016	0.463248
Itch	2.604015834	0.716919	0.275313
Nup98	2.603398743	1.29171	0.496163
Hcfc1	2.603365023	0.649015	0.249298
Gm2a	2.602669008	1.03543	0.397834
Srp72	2.600578888	1.116434	0.429302
Isy1	2.598795376	1.158508	0.445787
Kansl3	2.593111768	1.216365	0.469075
Arid1a	2.591326657	0.860952	0.332244
Myo10	2.591070418	0.880306	0.339746
Brd9	2.588035156	0.751354	0.290318
Gm3912	2.586909262	0.867323	0.335274
Cltc	2.586349847	0.910252	0.351944
Mllt10	2.585835097	0.588979	0.227771
Psme4	2.584925216	0.753749	0.291594
Glg1	2.584083403	0.83925	0.324777
Mapk1ip1l	2.583586822	0.36592	0.141633
Nfib	2.582144231	0.526779	0.204008
1810013L2	2.578913769	0.635853	0.246558
St8sia2	2.576220612	1.057515	0.410491
Xrn1	2.576100354	0.932763	0.362083
9930021J0	2.575664988	0.770207	0.299032
Phkb	2.569129347	1.171902	0.456148
Cspp1	2.567520262	1.02718	0.400067
Olfml1	2.567215008	1.256958	0.489619
Etnk1	2.565348682	0.96375	0.37568
Ptpn13	2.56385204	0.884076	0.344823
Tpr	2.559668979	0.642104	0.250854
Sdc3	2.559663712	0.839408	0.327937
Dmxl2	2.55742221	0.236758	0.092577
Kdm3a	2.556681939	1.020171	0.399022
Sox8	2.554384081	1.222357	0.478533
Usp19	2.553838376	1.129058	0.442102
Dpp3	2.550682524	0.831033	0.325808
Gm2012	2.543853157	0.656215	0.257961
Psmd1	2.541132028	1.243595	0.489386
Zc3h7a	2.539243162	0.6055	0.238457
Zfp84	2.53547364	1.196979	0.472093
Gareml	2.53195961	1.235656	0.488024
Eif3e	2.531751166	1.160188	0.458255
Shisa7	2.527632343	1.199201	0.474437
Cul4b	2.524422106	1.150407	0.455711
Nacc2	2.52326364	0.529122	0.209698
Acp6	2.519651406	1.233612	0.489596
Suco	2.516346441	0.996715	0.396096
Mrpl50	2.514261164	1.236567	0.491821

Bmpr1a	2.513068896	1.207834	0.480621
Morf4l2	2.509861991	1.070259	0.426422
Plcd4	2.508656781	0.440847	0.17573
C030037D(	2.50732275	1.136583	0.453305
Echdc1	2.500843994	1.058218	0.423144
Tcf4	2.498475959	0.950237	0.380327
Agpat3	2.49555803	0.618057	0.247663
Sgta	2.494982694	1.090357	0.43702
Rrbp1	2.494181526	0.714183	0.28634
Tlk2	2.493922887	1.040665	0.41728
Negr1	2.492776181	0.532407	0.21358
Mfap1b	2.488179656	1.058723	0.425501
Rapgef3	2.486831513	1.109395	0.446108
Tnfaip1	2.486462367	1.239784	0.498613
Clec2e	2.483826914	0.75796	0.305158
Hist2h3c1	2.483477187	0.851142	0.342722
Gnai3	2.483061345	0.985371	0.396837
Slu7	2.479167529	1.055923	0.425918
Prelp	2.47841869	1.1257	0.454201
Rab14	2.478348455	1.000125	0.403545
Ric1	2.474282787	1.181391	0.477468
Acaca	2.473501278	0.53326	0.215589
Tex9	2.471374953	1.061285	0.429431
Gm16026	2.47010169	1.2071	0.488684
Brd2	2.467232816	0.557838	0.226098
Nr2c2	2.466704723	0.922956	0.374166
Ehmt1	2.464849435	0.955471	0.387639
Acad9	2.463334611	1.177036	0.477822
Sema6a	2.462561651	1.21079	0.491679
Bpgm	2.459470915	1.123024	0.456612
Kdm5b	2.459373783	0.813315	0.3307
Pld2	2.455603224	0.505678	0.205928
Add1	2.45081958	0.758222	0.309375
Wac	2.450602332	0.946455	0.386213
Fam208a	2.450339917	1.166417	0.476023
Fkbp8	2.446468502	1.028312	0.420325
Lhfp	2.445816107	0.803673	0.328591
Tnrc6a	2.443973282	0.375853	0.153788
Dnaja1	2.443357079	0.895711	0.36659
Tet2	2.443139567	0.337059	0.137962
Tmem87a	2.443033325	1.119507	0.458245
Secisbp2l	2.441635342	0.938823	0.384506
Atf6	2.43762572	0.589107	0.241672
Cdk19	2.436630374	0.763147	0.313198
Ggps1	2.436561448	0.955266	0.392055
Mfap3l	2.435520737	0.992031	0.407318
Sec24b	2.430440613	0.517242	0.212818

Fam120a	2.429968661	0.690463	0.284145
Taok1	2.427912557	0.709582	0.29226
Trp53bp1	2.423889996	0.858793	0.354304
Wapal	2.42131075	0.789802	0.326188
Srsf7	2.419636524	0.899363	0.371694
Aup1	2.419382488	1.055646	0.436329
Ppp2r2a	2.417107875	0.936352	0.387385
Cdc42bpa	2.414733752	0.610646	0.252884
Clpx	2.41387831	0.811651	0.336243
Tmed10	2.412887257	0.964625	0.39978
BC003331	2.411881258	0.856822	0.35525
Gm9881	2.409611585	1.114773	0.462636
Fh1	2.406091718	1.156151	0.48051
Gpx4	2.403740586	1.164839	0.484594
Exoc1	2.402619796	0.905777	0.376996
Ppp1r2	2.401475388	1.125783	0.468788
Pltp	2.400308747	0.615453	0.256406
Tfg	2.398308414	0.977563	0.407605
Fam168a	2.3957432	0.713198	0.297694
Ythdc1	2.394875518	0.569319	0.237724
Muc3	2.391893198	1.033488	0.43208
Tox	2.390667745	0.949023	0.39697
Wwc1	2.389405431	0.783768	0.328018
Ptprd	2.386349317	0.418657	0.175438
Smarca2	2.386188053	0.564947	0.236757
Dync1h1	2.377929235	0.738904	0.310734
Usp15	2.373920468	0.9619	0.405195
5430403G:	2.367690298	0.897455	0.379042
Snx5	2.367497554	0.940091	0.397082
Med23	2.366480677	0.761618	0.321836
Tspan5	2.361795594	0.698058	0.295562
Zeb2	2.361373543	0.387328	0.164026
Abi1	2.360861387	0.575706	0.243854
Opa3	2.360753456	0.735911	0.311727
Erbb4	2.360590551	0.27276	0.115547
Dido1	2.359427848	0.661347	0.2803
Metap2	2.3586411	0.541371	0.229527
Actr3b	2.358502769	1.135593	0.481489
Asap2	2.353409488	0.724328	0.307778
Tnfrsf19	2.352579684	0.974243	0.414117
Gnai2	2.349918051	1.00171	0.426275
Mapk8ip3	2.34402857	1.01127	0.431424
Tial1	2.335927109	0.898012	0.384435
Pik3ip1	2.334618177	1.085637	0.465017
Tubb4b	2.334311389	0.783847	0.335794
Srcap_1	2.332677349	0.69478	0.297846
Kansl1l	2.331490883	0.709126	0.304151

Klf3	2.327872109	0.910298	0.391043
Cisd2	2.326808863	0.933332	0.401121
Rbm26	2.324943571	0.944968	0.406448
Agfg1	2.322593649	0.819641	0.352899
Tbp	2.316066338	1.157578	0.499804
Usp31	2.315066559	0.865931	0.374041
Scaf4	2.31391798	0.795856	0.343943
Rnf19a	2.313859667	1.059358	0.457832
Creb3l2	2.313386914	1.092406	0.472211
Cdc42bpb	2.310388333	1.054866	0.456575
Pcm1	2.310223916	0.68501	0.296512
Ddb2	2.309605166	0.735031	0.318249
Dhx15	2.308766274	0.357733	0.154946
D5Ertd579	2.306485727	0.193796	0.084022
Zbp1	2.305969708	0.906321	0.393032
Thrap3	2.305166166	1.105816	0.479712
BC005561	2.302717066	0.44275	0.192273
Spen	2.300110307	1.070536	0.465428
Zhx3	2.299594402	0.446724	0.194262
Slc20a1	2.297605189	0.501592	0.218311
Sort1	2.296400531	1.003552	0.437011
AI849053	2.291690007	1.063104	0.463895
Hint3	2.291430016	1.084277	0.473188
Snx25	2.290069307	0.944652	0.412499
Mink1	2.288393716	0.726377	0.317418
Fam98b	2.288289507	1.060012	0.463234
Zfp955b	2.287750826	0.936886	0.409523
Snw1	2.286344708	1.069638	0.467838
Spred2	2.285071824	0.84415	0.36942
AW54987	2.283492115	0.630089	0.275932
Ndrg3	2.282924399	0.971792	0.425678
Stk38l	2.279458372	0.895455	0.392837
Ppp2r3a	2.279313463	0.678081	0.297493
Stx17	2.277162609	1.123931	0.493566
Midn	2.274748783	1.067353	0.469218
Pias3	2.274380615	0.979305	0.430581
Gdap1l1	2.267028173	0.900891	0.397388
Zfp568	2.266809439	1.001182	0.44167
Dennd6a	2.265951876	0.463108	0.204377
Smox	2.263353678	1.055243	0.46623
Smg6	2.262238716	0.626502	0.276939
Map3k19	2.258237289	0.999189	0.442464
Heyl	2.258070393	1.111177	0.492092
Trio	2.255770226	0.828056	0.367084
Tra2b	2.255352856	0.963759	0.427321
Mut	2.247323882	0.516769	0.229949
Cmip	2.246321051	0.808475	0.359911

BC030336	2.246123643	0.974838	0.434009
B230307C2	2.242277308	1.091789	0.486911
Fer	2.241241423	0.920659	0.410781
Ppil4	2.240470831	0.887781	0.396247
Htatsf1	2.239570204	0.832689	0.371808
Gm5934	2.23931691	0.632958	0.282657
Tnpo1	2.238252928	1.064052	0.475394
Zfp609	2.235260819	0.637169	0.285054
Dleu2	2.235231939	0.929151	0.415684
Ermp1	2.234788164	0.767855	0.343592
Edem3	2.232566092	0.829943	0.371744
Sypl	2.232144307	0.712065	0.319005
Fam20a	2.231944981	0.85613	0.38358
Tulp4	2.231197289	0.542346	0.243074
R3hdm1	2.22638313	0.603473	0.271055
Megf10	2.226304786	0.614356	0.275953
Chst1	2.224946623	0.720056	0.323629
Clstn1	2.222234705	0.493206	0.221941
Cdk11b	2.221356294	0.59493	0.267823
Zfp462	2.221342534	0.468882	0.21108
Agpat5	2.218156299	0.565915	0.255128
Cflar	2.217279059	1.018157	0.459192
Ncl	2.21534968	0.926866	0.418384
Clock	2.215209858	0.403528	0.182162
Ssbp2	2.215019886	0.679328	0.306692
Cit	2.213603747	0.44228	0.199801
Tmed5	2.213526461	0.574428	0.259508
Bmp7	2.21320164	0.851637	0.384799
Dlg5	2.210968939	0.769394	0.347989
Crtc3	2.210360556	1.022142	0.462432
Abhd4	2.208309333	0.678466	0.307233
Vmn2r-ps5	2.203234938	0.61788	0.280442
Tmem220	2.202034959	0.809061	0.367415
Hspb11	2.199120693	0.748916	0.340552
Slc20a2	2.197196421	0.767794	0.349443
Gpr19	2.19686103	0.69635	0.316975
Gm20914	2.189386579	0.809854	0.3699
Dctn2	2.187765243	1.032488	0.471937
Nufip2	2.182849034	0.950634	0.435502
Pisd-ps1	2.181348164	1.005262	0.460844
Nap1l1	2.178575636	0.467198	0.214451
Golga4	2.178478363	0.819176	0.376031
Jarid2	2.177185908	0.81972	0.376504
Ttyh3	2.175846453	0.524741	0.241166
Gmfb	2.175620614	1.030984	0.473881
B3galt5	2.173002286	1.062023	0.488735
Abhd14b	2.169902002	1.040384	0.479461

Nrf1	2.168046049	0.795768	0.367044
Fbxw11	2.163012703	0.70241	0.324737
Ilkap	2.162629989	0.918659	0.424788
Serbp1	2.158903037	0.573229	0.265518
Mapk10	2.158709303	0.509379	0.235965
Gpr155	2.157637185	1.04215	0.483005
Atad2b	2.15558911	0.905949	0.420279
Ankrd12	2.153927274	0.518067	0.240522
Eif5b	2.153550618	0.638916	0.29668
4932438A1	2.151529364	0.442276	0.205563
Abi2	2.150881537	0.860652	0.400139
Rabep1	2.14981798	0.54043	0.251384
Dhcr24	2.148891956	0.972713	0.452658
Aff1	2.148668304	0.856387	0.398566
Olfr681	2.148142776	0.975273	0.454007
Dctn6	2.14770198	1.058144	0.492687
Tns3	2.147065865	0.815447	0.379796
Max	2.146777826	0.766354	0.356979
Gng12	2.146567758	0.652377	0.303916
Hnrnpf	2.146016013	1.056846	0.492469
Rala	2.145761589	0.805695	0.375482
Anapc16	2.143764358	0.893573	0.416824
Ctnnb1	2.142085094	0.556772	0.25992
Kdm4c	2.140013703	0.901794	0.421397
Pfdn5	2.138202445	0.844751	0.395075
Pcdh19	2.137688555	0.989241	0.462762
Tfdp1	2.135708421	1.042302	0.488036
Lrrc4b	2.135159801	0.91156	0.426928
Smc5	2.1350967	0.871725	0.408284
Dpf2	2.133328033	1.047382	0.490961
Gm904	2.131218349	0.926796	0.434867
Rftn2	2.130671246	0.705386	0.331063
Tspan31	2.130174079	0.615141	0.288775
Gltscr1	2.128251802	0.618163	0.290456
Pla2g16	2.128093512	0.719609	0.338147
Arhgap35	2.127078622	0.987248	0.464133
Larp1	2.126923081	1.006876	0.473396
Gm16630	2.124577011	0.860579	0.405059
Cachd1	2.123418688	1.017281	0.479077
Ccnk	2.123134056	0.867917	0.408791
Gab1	2.122183442	0.687806	0.324103
Slc25a36	2.121830083	0.696627	0.328314
Lbh	2.119420504	0.9386	0.442857
Cers2	2.11797769	0.493391	0.232954
Akap6	2.116385943	0.51349	0.242626
Cops3	2.116310012	0.796591	0.376406
Birc6	2.115636839	0.731958	0.345975

Xirp2	2.113445142	0.826009	0.390835
Phip	2.111734721	0.816738	0.386762
Avl9	2.108306742	0.751317	0.35636
Sik3	2.106046917	0.906639	0.430493
Ppia	2.105262779	0.681442	0.323685
Dynll1	2.103369529	0.966515	0.459508
Spns1	2.101874706	0.61637	0.293248
Eif4g1	2.100761513	0.651444	0.310099
Golgb1	2.099144664	0.900405	0.428939
Pcbp2	2.098174255	1.048583	0.49976
Cul4a	2.097576996	0.964792	0.459955
Dnm2	2.09448909	0.610216	0.291344
Arhgap12	2.092480008	0.831713	0.397477
Med15	2.091860907	0.676511	0.323401
Orc4	2.089753821	0.408874	0.195656
Rnf220	2.088597508	0.856651	0.410156
Cds2	2.087908902	0.665088	0.318543
Ighv1-37	2.08319153	0.930214	0.446533
Xbp1	2.081087048	0.260944	0.125388
Cic	2.080143318	0.84675	0.407063
Atg2b	2.077967525	0.824144	0.396611
Scaf8	2.077062367	0.911256	0.438724
Larp4	2.076194233	0.809938	0.390107
Strn3	2.076129082	0.815093	0.392602
Zfp788	2.074682322	0.474253	0.228591
Hipk1	2.074436311	0.718942	0.346572
Atrn	2.07283795	0.461553	0.222667
Usp16	2.071616908	0.521178	0.25158
Zfr	2.069141962	0.655911	0.316997
Arhgef26	2.067991795	0.747104	0.36127
Arcn1	2.066839256	0.805642	0.389794
Usp53	2.066417446	0.794357	0.384413
Mecp2	2.06627869	0.817367	0.395574
Usp38	2.065470918	0.955141	0.462432
Pkp4	2.064939276	0.369341	0.178863
Sec16a	2.064499898	0.632842	0.306535
Tpgs2	2.060817399	0.95921	0.465451
Agrn	2.060564926	0.627332	0.304446
Prrc2b	2.059087515	0.362616	0.176105
Rdh5	2.057306636	0.949824	0.461683
Atxn7	2.054833426	0.896646	0.436359
Atn1	2.053048742	0.88699	0.432035
Cluh	2.0528555	0.969914	0.472471
Arhgap42	2.050850359	0.670186	0.326784
Nptn	2.050749697	0.59581	0.290533
Pigb	2.048193746	0.888843	0.433964
Cpsf6	2.047458097	0.726766	0.35496

Gpi1	2.047282374	0.950936	0.464487
Dctn5	2.047133647	0.934108	0.456301
Dus3l	2.047120668	0.851899	0.416145
5031434O	2.045542181	0.75248	0.367863
Calu	2.043379728	0.849946	0.415951
Camk1d	2.042767534	0.52818	0.258561
Kpna6	2.042695111	0.920559	0.450659
Fyco1	2.042333131	0.941064	0.460779
Sidt2	2.04163696	0.93271	0.456844
Nol4	2.040554176	0.743884	0.36455
Tnrc6b	2.040173486	0.729764	0.357697
Tsc1	2.038502919	0.56629	0.277797
Dcaf8	2.038416981	0.473809	0.23244
Nup93	2.037667753	0.525934	0.258106
Tbce	2.036994441	0.868908	0.426564
Ulk1	2.035606758	0.755587	0.371185
Rc3h1	2.035164454	0.649888	0.319329
Smek2	2.0303046	0.682374	0.336094
Hnrnpul2	2.028765064	0.811162	0.399831
Pum1	2.027742773	0.92253	0.454954
Snx13	2.02756606	0.940019	0.46362
Bsg	2.025468488	0.765424	0.3779
Rcbtb1	2.024780642	0.963629	0.475918
Rpl31	2.021957	0.956906	0.473257
Dnah7b	2.021017707	0.835614	0.413462
Trpm7	2.020120813	0.623292	0.308542
Peli2	2.016613921	0.621646	0.308262
Nebl	2.011401022	0.362716	0.18033
Eif2a	2.008432422	0.75334	0.375088
Specc1	2.007034707	0.850825	0.423921
Reep3	2.006248873	0.717514	0.35764
Ctsf	2.003635811	0.914417	0.456379
Atp5o	2.003562986	0.969025	0.483651
